# Genetic Diversity of Staphylocoagulase Genes (*coa*): Insight into the Evolution of Variable Chromosomal Virulence Factors in *Staphylococcus aureus*

**DOI:** 10.1371/journal.pone.0005714

**Published:** 2009-05-27

**Authors:** Shinya Watanabe, Teruyo Ito, Takashi Sasaki, Shanshuang Li, Ikuo Uchiyama, Kozue Kishii, Ken Kikuchi, Robert Leo Skov, Keiichi Hiramatsu

**Affiliations:** 1 Department of Infection Control Science, Graduate School of Medicine, Juntendo University, Bunkyo-ku, Tokyo, Japan; 2 Department of Bacteriology, School of Medicine, Juntendo University, Bunkyo-ku, Tokyo, Japan; 3 National Institute for Basic Biology, National Institutes of Natural Sciences, Myodaiji, Okazaki, Japan; 4 National Center for Antimicrobials and Infection Control, Statens Serum Institut, Copenhagen, Denmark; Baylor College of Medicine, United States of America

## Abstract

**Background:**

The production of staphylocoagulase (SC) causing the plasma coagulation is one of the important characteristics of *Staphylococcus aureus*. Although SCs have been classified into 10 serotypes based on the differences in the antigenicity, genetic bases for their diversities and relatedness to chromosome types are poorly understood.

**Methodology/Principal Findings:**

We compared the nucleotide sequences of 105 SC genes (*coa*), 59 of which were determined in this study. D1 regions, which contain prothrombin-activating and -binding domains and are presumed to be the binding site of each type-specific antiserum, were classified into twelve clusters having more than 90% nucleotide identities, resulting to create two novel SC types, XI and XII, in addition to extant 10 types. Nine of the twelve SC types were further subdivided into subtypes based on the differences of the D2 or the central regions. The phylogenetical relations of the D1 regions did not correlate exactly with either one of *agr* types and multilocus sequence types (STs). In addition, genetic analysis showed that recombination events have occurred in and around *coa*. So far tested, STs of 126 *S. aureus* strains correspond to the combination of SC type and *agr* type except for the cases of CC1 and CC8, which contained two and three different SC types, respectively.

**Conclusion:**

The data suggested that the evolution of *coa* was not monophyletic in the species. Chromosomal recombination had occurred at *coa* and *agr* loci, resulting in the carriage of the combinations of allotypically different important virulence determinants in staphylococcal chromosome.

## Introduction

*Staphylococcus aureus* is a persistent resident of the nasal membrane and skin of warm-blooded animals, and a major causative agent of hospital and community-associated infections. Staphylocoagulase (SC) that causes coagulation of plasma is one of the extracellular virulence factors produced by *S. aureus* strains, and is regarded as a marker for discriminating *S. aureus* from other less pathogenetic staphylococci called as coagulase-negative staphylococci.

**Figure 1:**
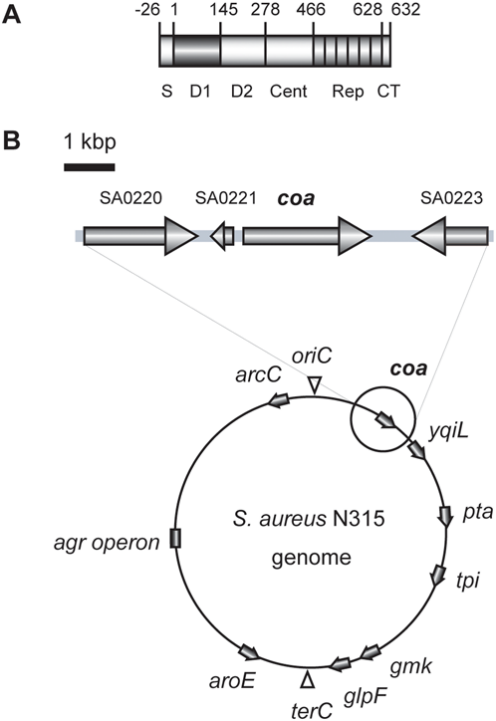
Stracture of SC and its gene locus. A. Domain organization of SC in *S. aureus* N315. S, signal sequence; D1, D1 region; D2, D2 region; Cent, central region, Rep, repeat region; CT, C-terminal region. B. Organization of the *coa*-flanking region and the loci of *coa*, *agr* operon and MLST genes in *S. aureus* N315 genome.

SC causes the coagulation of plasma without the usual proteolytic cleavages caused by factor Xa. SC binds to prothrombin and the complex of SC and prothrombin induces plasma coagulation by converting fibrinogen into fibrin [1], [2]. Variations in SCs have been noticed as the differences in the antigenicity, and SCs have been classified into 10 serotypes by inhibition test of their clotting activity using type-specific antibodies against each serotype [3], [4]. We previously reported the nucleotide sequences of the SC genes (*coa*) of all 10 extant serotypes [5]. Structural comparisons of the deduced amino acid sequences of 10 *coa* showed that SCs were composed of six fundamental segments: signal sequence at N-terminus, D1 region, D2 region, central region, 27 amino-acid repeat region and C-terminal sequence of 5 amino acids (figure 1). SC binds to prothorombin via the D1, which contains N-terminal prothorombin-activating domain, and the D2 region [6]. Comparisons of nucleotide sequences as well as their deduced amino acid sequences of *coa* of 10 serotypes showed the D1 and the D2 regions were rather diverse, whereas the central regions were relatively conserved. Since identities of both nucleotide and amino acid in the D2 regions were higher than those in the D1 regions, it has been suggested that the D1 region might be responsible for the antibody recognition site for type specific antiserum [5].

In Japan, SC serotyping has been applied to epidemiological study of *S. aureus* isolates. However, this method has not been widely used in other countries. It seemed that it was regarded as time-consuming and laborious method or that researchers were unaware of the method. Therefore, we recently developed multiplex PCRs (M-PCRs) to classify SC types simply and rapidly [7]. So far as tested, the results of the M-PCRs correlated well to those of serotyping.

Accessory gene regulator (*agr*) operon, well known regulatory system in *S. aureus* strains, is composed of a large set of genes, *agrA*, *agrC*, *agrD*, *agrB*, and RNAIII. Since variation exists in the regions from *agrC* to *agrB*, *agr* typing can be done by either PCR or by determining nucleotide sequences of *agrD*. Four *agr* types are reported, and used for epidemiological classification of *S. aureus* isolates [8]. A more extensive typing method, multilocus sequence typing (MLST), has been widely used, which assigns every strain with a sequence type (ST) [9]. By using eBURST program, phylogenetically related STs can be grouped as a clonal complex (CC) [10].

In this paper, we studied correlation of the two typing methods with the SC typing by using 105 *S. aureus* strains with their *coa* sequenced and 21 strains whose SC types were determined by the M-PCRs and serotyping. Analysis for recombination events among *coa* with their flanking regions suggested that horizontal genetic transfer in and around *coa* have occurred among *S. aureus* stains.

## Materials and Methods

### Bacterial strains and culture conditions

A total of 126 methicillin-resistant and susceptible *S. aureus* strains (MRSA and MSSA) from various categories were used in this study including 70 MRSA (64 from humans and 6 from cats) and 40 methicillin-susceptible *S. aureus* (MSSA) strains (11 from humans and 29 from cats and cows) (table S1) [3], [4], [7], [11]–[19]. *S. aureus* strains were cultivated at 37°C in tryptic soy broth or on tryptic soy agar (Becton Dickinson CO., Ltd.), and stored at −80°C in 50% glycerol.

We also used the nucleotide sequences of two *coa* partially-sequenced strains isolated in Hokkaido in Japan [20] and of fourteen whole genome-sequenced strains [21]–[31] obtained from the DDBJ/EMBL/GenBank databases.

### SC typing of staphylocoagulase

Serotying: The Serotypes of SCs were determined by the inhibition test for coagulation of plasma using commercially available specific antibodies to type I to VIII SCs of *S. aureus* (Denka Seiken Co., Ltd., Tokyo, Japan). We did not determine type IX and type X SCs since antisera to the SC types were not commercially available [5].

M-PCRs: The SC types were determined by M-PCRs consisting of two sets of primers identifying types I–VIII *coa* [7]. M-PCR set A and set B contained primers for identifying the *coa* of type III, IV, VII and VIII, and *femA* as an internal positive and primers identifying the *coa* of type I, II, V, and VI, and *femA*, respectively.

### DNA preparation

Chromosomal DNA was extracted using Isoprant (Nippon Gene Co,. Ltd., Tokyo, Japan) [5]. 800 µL of the overnight culture in tryptic soy broth was poured into a tube, and cells were collected by centrifugation. The pellet was re-suspended with 400 µL of SMM buffer (0.5 M sucrose, 0.1 M disodium maleate, 0.002 M MgCl_2_: pH6.5), 20 µL of 2 mg/ml lysostaphin (Wako Pure Chemical Industries, Ltd, Osaka, Japan) was added, and then the mixture was left at 37°C for more than 20 min until protoplasts were formed. The protoplasts were collected by centrifugation, and their chromosomal DNAs were extracted by Isoparant as recommended by the manufacture.

### DNA sequencing of *coa*

*coa* was amplified by PCR using chromosomal DNA as a template. DNA fragments were amplified using primer sets: coa-F-1 (5′ -TAATGTAGATTGGGCAATTACA-3′) and coa2 (5′- ATGCTTTAATTCAGTTAGAAGC-3′). The reaction mixtures consisted of 50-µL total volume solutions containing 10 ng of genomic DNA, 5 pmol of each primer, 0.2 U of Ex *Taq* DNA polymerase (Takara Bio Inc., Shiga, Japan), 5 µL of Ex *Taq* Buffer and 10 mM of each deoxynucleotide triphosphate. Amplification was performed with Thermal Cycler Dice (Takara Bio Inc.), and parameters were 30 cycles of 30 s at 94°C, 1 min at 55°C and 2 min at 72°C, and hold at 4°C. The PCR products were purified using a High Pure PCR product purification kit (Roche Molecular System Inc.). Nucleotide sequencing was carried out using BigDye Terminator version 3.1 Cycle Sequencing Kit (Applied Biosystems, CA, USA) and 3730 DNA Analyzer (Applied Biosystems, CA, USA and Hitachi, Ltd., Tokyo Japan) according to the manufacturer's instructions. The flanking regions of *coa* were determined as described previously [5].

### MLST

MLST was preformed as described previously [9]. CC was determined by eBURST analysis [10]. It was defined as a group in which the STs were identical in sequence at six of seven MLST genes (*arcC*, *aroE*, *glpF*, *gmk*, *pta*, *tpi* and *yqiL*) to at least one other ST in the group.

### *agr* typing

*agr* types were determined using two sets of M-PCRs reported previously [8].

### Bioinformatic analysis

Genetic analysis was mainly carried out using programs in GENETYX-MAC Version 13.0.3 (GENETYX Corporation, Tokyo, Japan).The nucleotide sequences of *coa* or concatenated fragments of MLST genes were aligned using the clustalW Version 1.83 program with 1000 times bootstrapping [32] and neighbor-joining analysis was carried out with Tajima-Nei parameter model. Phylogenetic trees were visualized with TreeView X program [33]. Recombinations in and around *coa* were detected using the RDP3 Beta 34 software [34]. We used six automated recombination detection methods, RDP [35], Geneconv [36], Bootscanning [37], Maximum Chi Square (MaxChi) [38], Chimaera [39], and Sister Scanning (SiScan) [40]. Results were verified by visual inspection.

### Nucleotide sequence accession numbers

The nucleotide sequences of *coa* and their flanking sequences used in this study have been deposited in the DDBJ/EMBL/GenBank databases under accession numbers as follows: AB436972-AB436988 (17 entries), AB437138, AB488498-AB488510 (13 entries), and AB489873- AB489901 (29 entries).

## Results

### Common structures shared among 103 SCs

To understand the overall structures as well as their diversities of *coa* in *S. aureus*, we listed 103 strains and conducted experimental characterization of SCs of 100 strains by serotyping and M-PCRs except for three strains of which we could use sequence data only. By serotyping, 78 strains were classified into either one of eight serotypes, but 7 strains could not be classified into one of eight serotypes and 15 strains could not be tested since coagulation of plasma could not be observed for up to 48 h incubation (table S1). With M-PCRs, *coa* of 88 strains were classified into one of eight types, leaving that of 12 strains still unclassifiable. We determined nucleotide sequences of *coa* of 59 strains that contained SC-nontypeable strains by both serotyping and M-PCRs, and that were chosen mostly based on genetic backgrounds distinct from 10 reference strains of SC types and other *coa*-sequenced strains. The structures of these 59 *coa* were analyzed together with the extant 44 *coa*: 30 of *coa*-sequenced strains and 14 of whole genome-sequenced stains. The overall structures of *coa* and other characteristics of the 103 *coa*-sequenced strains including *agr* types and STs are shown in figure 1 and table S1, respectively.

All *coa* are composed of the 6 regions. The 5′ end of 78-bp and the 3′ end of 18-bp are identical among all the genes except for a *coa* that carried single nucleotide silent mutation in the 5′ end. They carried D1, D2, and central regions, other than the case of strain M, of which *coa* had a 140 amino acids deletion spanning from the D2 region to the central region. Although all of them carried 81-bp tandem repeats at the 3′ terminus, the numbers of repeats were diverse ranging from one (TSCC26) to nine (NVAU02070, NVAU02080 and Ku). The sizes of *coa* ranged from the smallest one, 1404 bp (strain M) to the largest one, 2280 bp (Ku). Except for *coa* of strain M, the differences in sizes were mostly due to the number of 81-bp tandem repeats.

### SC types based on the differences among the D1 regions

**Figure 2:**
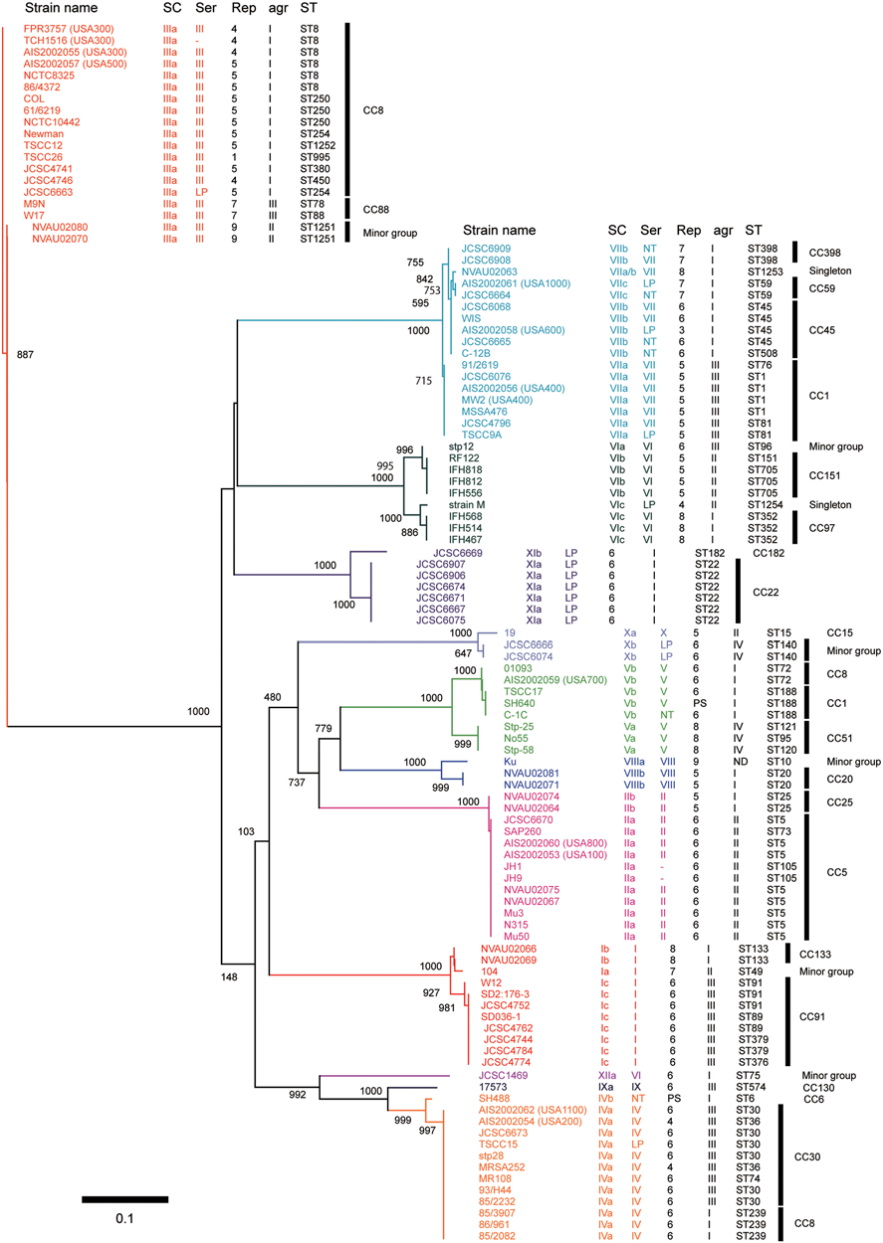
Phylogenetic relationship among the nucleotide sequences of the D1 regions of 105 *coa*. The Neighbor-Joining (NJ) tree was constructed using Clustral W and TreeView. The numbers at nodes refer to bootstrap replicates out of 1000 that support the node. Abbreviations are as follows: SC, the type of stapylocoagulase; Ser, serotype of staphylocoagulase; Rep, a number of 81-bp tandem-repeat units of *coa*; *agr*, *agr* type; ST, multilocus sequence type; LP in Serotype section, Staphylocoagulase production was too low to clot serum within 48 hr; NT in Serotype section, Non typable; “-” in Serotype section, Not tested; PS in Rep section, Partially sequenced; ND in *agr* section, Not detected by M-PCR for *agr*.

Historically, SCs have been classified into 10 serotypes based on the differences in antigenicity by inhibition test using type-specific antibodies against each type of staphylocoagulase proteins. Since our previous study suggested the D1 regions might be the major antibody recognition site for type-specific antibodies [5], we conducted nucleotide and amino acid sequence comparison among 103 genes and 2 partially-sequenced *coa*. Phylogenetic trees were created to compare the relationship between serotypes and nucleotide diversities in the D1 regions (figure 2) as well as amino acid diversities (data not shown).

The D1 regions of the 105 *coa* were classified into 12 major clusters and those belonging to each cluster showed nucleotide identities with more than 90% (figure 2). Since each one of 10 clusters contained one of the 10 SC reference strains, it was inferred that these 10 clusters represented extant 10 serotypes. Consequently, we regarded other two additional clusters as new types and designated them as types XI and type XII SC, respectively. As we describe bellow, a group including seven strains with ST22 or ST182 and with *agr* type I had type XI SC, and one strain with ST75 and *agr* type I had type XII SC. The average of nucleotide identity among the *coa* D1 regions of all the 12 SC types was 67.1%, ranging from the highest (89.0%, type IV vs type IX) to the lowest (58.8%, type III vs type IX) (table 1).

**Table 1:** Identities of nucleotide sequences of D1 regions of *coa* and their deduced amino acid sequences among 12 SC types.

strain name	SC type	Ia	IIa	IIIa	IVa	Va	VIa	VIIa	VIIIa	IXa	Xa	XIa	XIIa
104	Ia		53.7%	51.7%	57.7%	56.4%	57.7%	57.0%	60.4%	58.4%	57.0%	50.3%	57.7%
N315	IIa	64.4%		51.0%	50.3%	59.0%	51.4%	51.7%	61.7%	51.0%	51.7%	60.3%	55.7%
NCTC8325	IIIa	63.8%	63.1%		53.0%	41.6%	48.3%	53.0%	45.0%	55.0%	44.3%	53.7%	55.7%
stp28	IVa	67.6%	64.2%	65.5%		54.4%	49.7%	50.3%	55.0%	85.2%	45.6%	48.3%	70.5%
No55	Va	65.8%	70.5%	58.8%	66.9%		54.7%	46.3%	66.4%	54.4%	52.3%	53.4%	53.0%
stp12	VIa	67.6%	67.7%	66.4%	64.7%	66.3%		54.4%	59.1%	52.3%	51.0%	58.8%	49.7%
MW2	VIIa	69.1%	61.7%	64.4%	63.3%	63.5%	68.8%		51.7%	48.3%	54.4%	51.0%	50.3%
Ku	VIIIa	71.1%	70.5%	62.5%	66.6%	72.9%	69.5%	65.5%		56.4%	57.0%	59.7%	55.0%
17573	IXa	70.3%	66.2%	67.0%	89.0%	66.2%	68.6%	63.7%	69.6%		47.7%	49.7%	73.8%
19	Xa	66.2%	67.0%	64.9%	61.6%	66.0%	66.1%	67.2%	70.0%	63.8%		53.7%	46.3%
JCSC6671	XIa	65.0%	70.4%	67.8%	62.2%	68.0%	71.2%	68.2%	69.3%	64.4%	69.6%		55.0%
JCSC1469	XIIa	67.6%	65.8%	64.9%	76.7%	68.2%	63.5%	63.3%	66.3%	77.8%	64.2%	65.3%	

Identities of nucleotide sequences of D1 regions of *coa* and their deduced amino acid sequences among 12 SC types.

Nucleotide identities are shown in cells in the bottom left half of the table and amino acid identities are shown in cells in the upper right half of the table.

### SC subtypes classified based on the differences in either D2 or central region

We previously reported that type VI SCs were classified into three subtypes (types VIa, VIb and VIc) based on the nucleotide differences in the D2 or the central regions [7]. Although the three subtypes of type VI SCs were highly homologous with nucleotide identities of more than 96.6% in the D1 region, they differed considerably in the D2 and the central region (figure 2 and table S2). If we adopt the criteria that an SC type should be assigned with more than 90% nucleotide identities of the D1 regions, and these SC types should be classified further into distinct subtypes with more than 90% nucleotide identities in both the D2 and the central region, nine of 12 SCs were further subdivided into several subtypes. Two SC types (I and VII) can thus be subdivided into three subtypes, and six SC types (II, IV, V, VIII, X and XI) into two subtypes (table S2). The comparisons of nucleotide identities of the D1, the D2, and the central regions of the different subtypes are shown in table S2.

### Phylogenetic relationship of *coa* flanking regions

**Figure 3:**
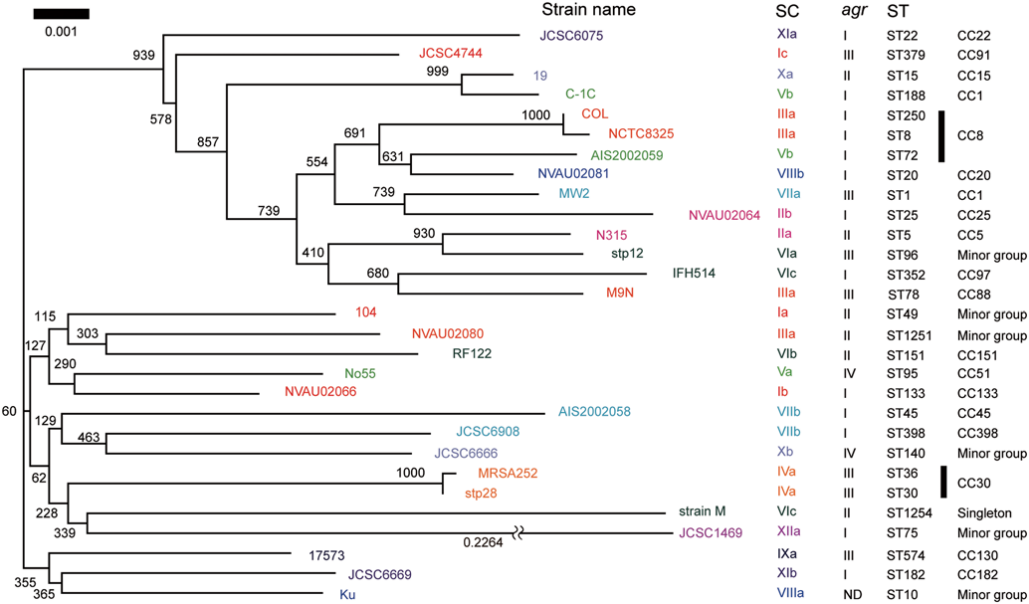
Phylogenetic relationship among the *coa* flanking regions of 29 *S. aureus* strains. The sequences of the regions spanning from ORF encoding hypothetical protein corresponding to glycerophosphodiester phosphodiesterase (SA0220) to ORF corresponding to acetyl-CoA acetyltransferase homologue (SA0223) apart from *coa* are used. The numbers at nodes refer to bootstrap replicates out of 1000 that support the node. Abbreviations are as follows: SC, stapylocoagulase (SC) type; *agr*, *agr* type; ST, multilocus sequence type; ND in *agr* section, Not detected by M-PCR for *agr*.

In order to know whether *coa* has coevolved with its surrounding regions, we sequenced *coa* flanking regions spanning from the region corresponding to SA0220 that is located before *coa* to the region corresponding to SA0223 that is located after *coa* (figure 1). A phylogenetic tree was created using the regions corresponding to SA0220–SA0223 of a total of 29 strains, from which the region corresponding to *coa* has been removed (figure 3). Twenty-nine strains represented different genotypes and SC types or subtypes (table S1).

In contrast to the phylogenetic tree of the D1 regions, in which strains of the same SC types clustered together, phylogenetic tree of the *coa* flanking regions showed that strains harboring the *coa* of the same SC types did not always belonged to the same cluster, but distributed in the different clusters. For example, the *coa* flanking regions of N315 (SC type IIa), was split from that of NVAU02064 (type IIb), but closely related to that of stp12 (type VIa). Instead, the *coa* flanking regions rather phylogenetically correlated to housekeeping genes examined by MLST as shown below, suggesting that *coa* evolved independently from other genes in the genome.

### Evidences for recombination among *coa* and its flanking regions

**Figure 4:**
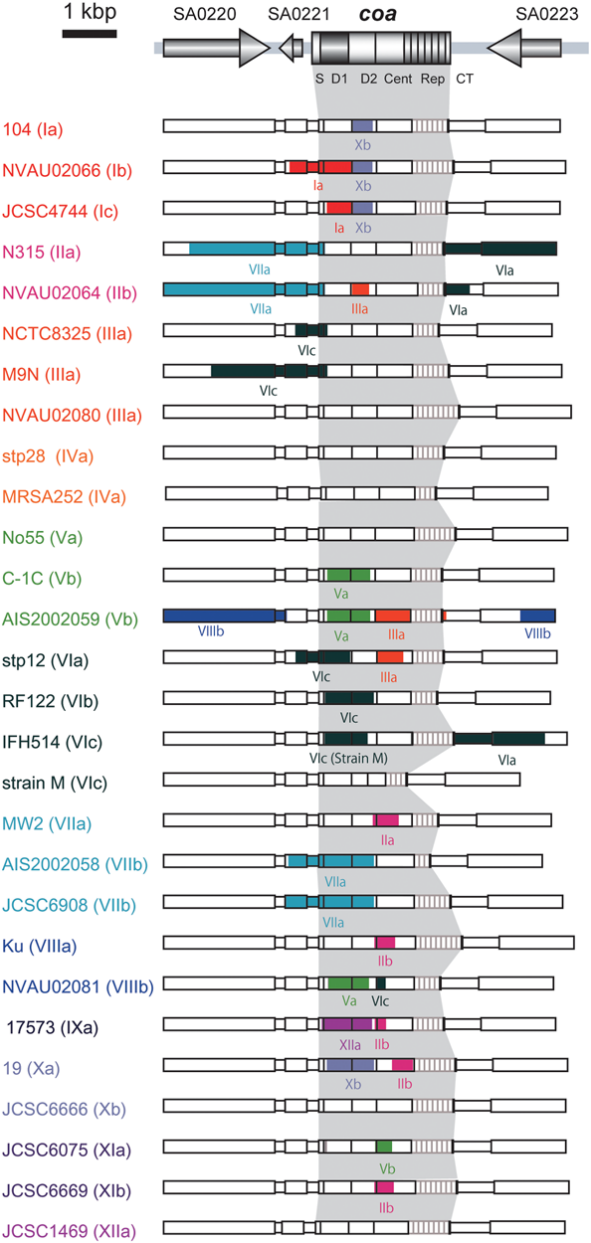
Predicted recombination sites identified in and around *coa* of 28 *S. aureus* strains. The nucleotide sequences from SA0220 to SA0223, from which the repeat sequences located at 3′ end of *coa* were removed, were used for this analysis. Recombination events and their breakpoints were detected by RDP3 Beta 34 software and those with significant P value<10^−5^ in at least three of six recombination-detecting programs are listed. Recombinant (daughter) sequences were indicated with the recombination sites derived from minor parent sequences highlighted in the color represented by each SC type.

The D1 region of *coa* presented much diversity, so did in a less degree the D2 and central regions. To know whether recombination played a role in the generation of the diversity, we sought evidence for recombination at the *coa* locus using RDP3 Beta 34 software. With the data set of 28 nucleotide sequences of the region spanning from SA0220 to SA0223, from which nucleotide sequences of the repeat regions were removed, 38 recombination events were predicted by the program. Among them, we listed 22 putative recombination events that were detected by more than 3 of six programs with statistical significance P<10^−5^ and were verified by visual inspection (figure 4). The DNA regions derived from a minor parent were identified by RDP3 and the data were confirmed by visual inspection, too. However, there are still possibilities that the relations between a minor parent and a recombinant might be replaced as suggested by the program.

**Figure 5:**
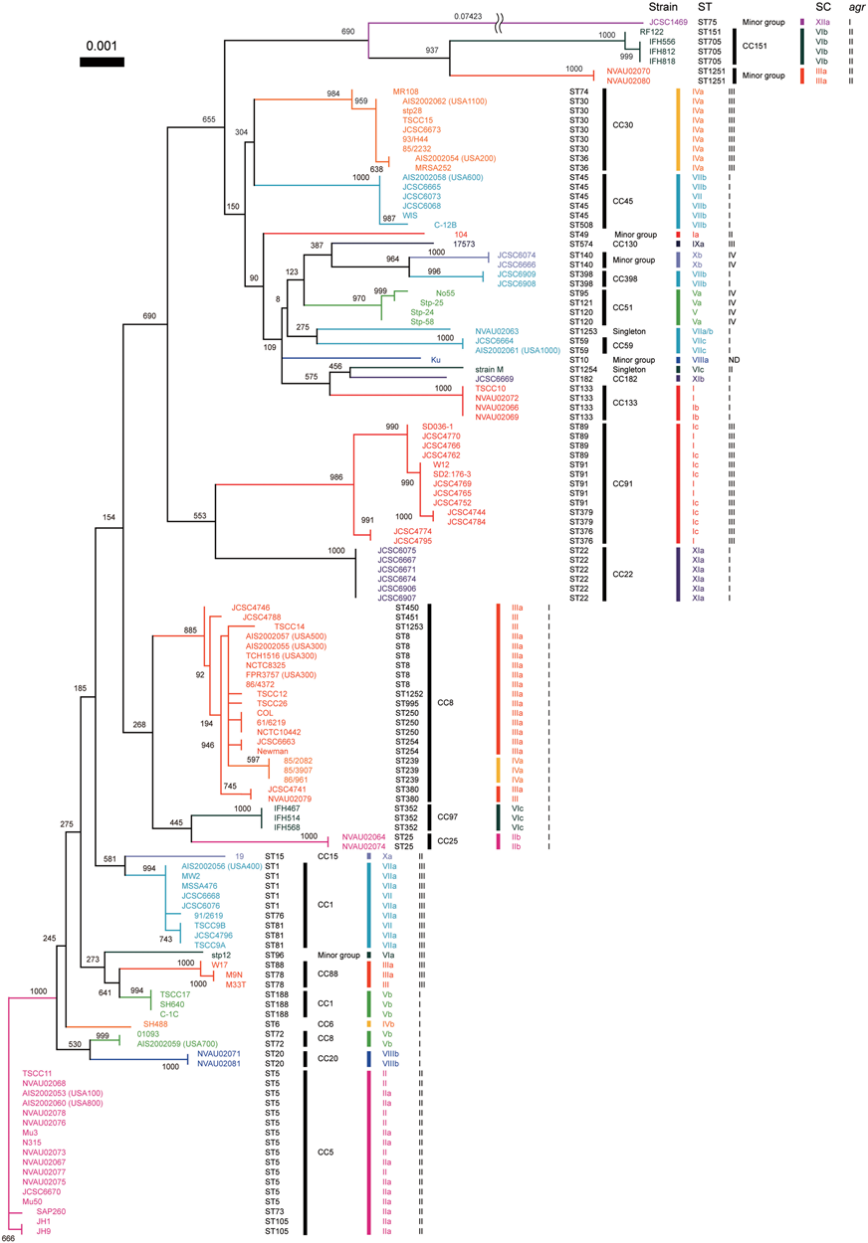
Phylogenetic relationship of core regions represented by concatenated sequences of seven housekeeping genes fragments used for MLST and their correlations to SC types and *agr* types. The NJ tree was constructed using Clustral W and visualized by TreeView. The numbers at nodes refer to bootstrap replicates out of 1000 that support the node. Abbreviations are as follows: SC, stapylocoagulase (SC) type; *agr*, *agr* type; ST, multilocus sequence type; ND in *agr* section, Not detected by M-PCR for *agr*. SC subtypes are indicated only in the cases of the *coa*-sequenced strains. SCs of the other strains, of which SC types were determined with M-PCR, were not subtyped.

The data clearly indicated that recombination have occurred in or around *coa* loci because all of them showed very low P values and were detected by most of the six recombination-detecting program. For example, the case of strain 17573 that showed the lowest P-value (6.93×10^−67^ in RDP, 2.52×10^−17^ in Geneconv, 2.76×10^−27^ in Bootscan, 1.34×10^−25^ in MaxChi, 7.33×10^−29^ in Chimaera and 2.96×10^−19^ in SiScan) among those of all predicted recombination events. The 796-bp region spanning from D1 region to D2 region in 17573 (SC type, IX) was replaced by that of JCSC1469 (SC type, XII). That is consistent with the fact that the D1 region of 17573 was relatively similar to that of JCSC1469 (figure 2) although MLST allele of 17573 was phylogenetically distinct from that of JCSC1469 (figure 5).

Most of the recombination events have occurred within *coa* loci. In some case, the recombination regions involving the D1 regions were replaced those of other background and the recombination seems to create new SC subtypes. For example, the 1005-bp region spanning from SA0221 to the D1 regions of *coa* of NVAU02066 (SC type Ib) and the 413-bp region involving the D1 region of JCSC4744 (SC type Ic) were replaced by that of 104 (SC type Ia). The recombination might contribute to the variation of *coa*. Similar events might have occurred in SC V, VI, VII and X strains (figure 4).

### The relationship between SC types and MLST allelic profiles

To gain an insight into the relatedness between SC types and MLST allelic profiles, we created a phylogenetic tree based on concatenated sequences of seven housekeeping genes used for MLST and compared their relations to SC types (figure 5). We used a total of 126 *S. aureus* strains (table S1), which were composed of 105 *coa*-sequenced strains described above and 21 strains whose SC types were determined by the M-PCRs [7].

Most of strains belonging to a given CC carried a *coa* of the same SC type except for the cases of two CCs. The strains belonging to CC1 carried two SC types, VIIa and Vb and those belonging to CC8 carried three SC types, IIIa, IVa and Vb. However, when looking at the relationship between ST and SC type, we found strains of a given ST type carried a specified SC type: ST8, type IIIa; ST239, type IVa; and ST72, Vb.

Although each cluster composed of a single CC was occupied by strains of the same SC type, strains of the same SC type but different subtype did not always show high phylogenetical relatedness to MLST genes (figure 5). In other words, some strains with a given D1-based SC type belonged to phylogenetically distinct CCs which were remote in the phylogenetic tree, e.g., SC type IIa belonged to CC5 and SC type IIb belonged to CC25. It suggested that multiple recombination including *coa* loci have occurred in *S. aureus* chromosome.

Strain JCSC1469, which was isolated in Australia [17], was considered to be an orphan isolate on the basis of the MLST tree (ST75), and did not cluster with any strains used in this study. It was assigned with a novel SC type XIIa, which was moderately related to SC type IX with 77.8% of the nucleotides identities in the D1 regions.

Comparing phylogenetic correlation between MLST genes and *coa* flanking regions, we found that they were relatively similar to each other. Some pairs of strains with different SC types were closely related in both trees of MLST genes and *coa* flanking regions, for example JCSC6075 (SC XIa) and JCSC4744 (SC Ic), AIS2002059 (SC Vb) and NVAU02081 (SC VIIIb), RF122 (SC VIb) and NVAU02080 (SC IIIa), JCSC6908 (SC VIIb) and JCSC6666 (SC Xb), and JCSC6669 (SC XIb) and Ku (SC VIIIa) (figure 3 and 5).

### The relationship among *agr* types, SC types and ST alleles

Furthermore, *agr* types of the 126 strains were determined by M-PCRs [8] to investigate relations among *agr* types, SC types and ST alleles. One hundred and twenty five strains could be classified into one of the four alleles of *agr*, leaving only one untypeable strain Ku. Similar to the case with SC typing, all the strains of a given CC were classified into the same *agr* group. Although each *agr* type was not assembled in the MLST tree, the data confirmed the previously described findings [41]. However, when we have examined the phylogenetic relationship between *agr* type and SC type, we found that strains belonging to a given *agr* type were classified into several SC types. In other words, a SC type did not always correlate to a specific *agr* type (figure 5 and Table S1). The *agr* type I strains harbored multiple SC types such as I–VIII, XI and XII. The *agr* type II strains contained such SC types as I, II, III VI and X, *agr* type III strains contained strains of SC types I, III, IV, VI, VII and IX, and *agr* type IV strains contained strains of SC types, V and X, respectively. Our data showed that a given CC belong to a SC type and an *agr* group so far tested, suggesting that a CC could be inferred by the combination of SC type and *agr* type that can be determined by PCRs rather easily.

## Discussion

We ascertained the common structure of *coa* by analysis of the 105 *coa* sequences. The clustering analysis of the D1 regions of the *coa* showed that they were classified into 12 clusters. Ten of the 12 clusters were correlated with extant 10 serotypes and the strains belonging to a cluster were assigned with a single serotype except for a case of strain JCSC1469, verifying that the D1 region contains the binding sites of antibodies used for serotyping. Identification of two novel types showed further variability of *coa*. SC types have been used as a marker in epidemiological study and the relationship between SC type and chromosome type have been suggested. In this study, we investigated relationship between SC type and ST supposed to represent the entire chromosome genotype by comparing the phylogenetic trees based on the *coa* sequences and the concatenated sequences of each MLST. The data indicated that there are significant correlation between the SC type and CC, suggesting that SC allele is a relatively stable characteristic in the chromosome of genetically-related strains which belong to a same CC.

To date, genomes of fourteen *S. aureus* strains have been sequenced [21]–[31]. Comparison of the genomes has revealed that the *S. aureus* genome consists of core genes and accessory genes [42], [43]. Most of the accessory genes were carried by mobile genetic elements, which have been acquired from intra- or interspecies genetic transfer. Such exchange of genetic information by horizontal gene transfer is a very important process for *S. aureus* to evolve its genome and adapt to environmental change [42]. Lindsay et al identified the core genes common to all strains by microarray analysis [43]. Some regions in the core genome are exceptionally variable in sequence between lineages and these genes were termed core-variable (CV) genes. Many CV genes encode virulence factors for extracellular or cell surface proteins such as *coa*, *fnbAB*, *ebh*, *sasAG* and *sdrDE*. The genes in *agr* cluster are also known as CV genes and they have four major variants [44], [45]. These CV genes are scattered throughout the genome and make up approximately 10 to 12% of any genome [43].

We analyzed two CV genes or gene allele, *coa* and *agr*, in this study. The nucleotide polymorphisms of these loci have been used for strain typing in *S. aureus*. However, we found here that they were not phylogenetically related to each other. By MLST, strains with the same SC type were scattered in the MLST tree (figure 5). This indicates that *coa* can be laterally transferred among different lineages. Similar to *coa*, all strains of a given CC were classified into the same *agr* group although each *agr* types was not assembled in the MLST tree. This is the same results reported previously that *agr* allele is correlated with CC [41].

*coa* and *agr* genes are located on the chromosome (figure 1) and neither of these genes reside on mobile genetic elements as judged from the genome sequences of 14 *S. aureus* strains. The nucleotide comparison of *coa* flanking regions suggested that *coa* and the flanking regions have not coevolved. Assuming that recombination events or mutations occurred more frequently at *coa* loci than at flanking regions, we tried to detect recombination event using RDP3 program with sets of 28 nucleotide sequences of approximately 5.7 kb DNA region. Among the predicted recombination events, we listed in figure 5 only events whose crossover points were clearly seen and in which relations between parents and daughter were indicated. The data show that recombination events have occurred at the region in or around *coa* loci. Identification of putative crossovers suggested that the recombination between *S. aureus* strains with different genetic backgrounds have occurred. We reported previously that *coa* belong to genomic islets and the direct repeat sequences identified immediate proximity of *coa* might have served as the recombination points to acquire an alternative *coa* allele [42]. However, in this study, such sequences could not be identified as recombination break points. Introduction of *coa* allele in the cell presumably by phage transduction or transformation and recombination between the two *coa* might have occurred in the past. The frequency of such the recombination might be not so frequent as genetic transfer caused by any mobile genomic elements, because SC type were conserved in the same CC except for a little exceptional case so far tested.

Most of all strains belonging to the same CC defined by MLST had the same SC type and *agr* type (figure 5). It indicates that determination of both SC and *agr* types could be predicted by the type of lineage of the *S. aureus* strains except for CC8 and CC1 strains. One of the reasons why this is seen in the CC8 group is because *coa* located in the large chromosome recombination regions, which was described previously [5], [46]. Combination of M-PCRs for *coa* and *agr* will be an useful strain typing method for understanding the changing epidemiology of MRSA infections and for evaluating the efficacy of outbreak intervention and prevention strategies in clinical setting.

In this study, we showed the differences in *coa* were observed mostly in the D1 regions among all SCs. Crystal structural analysis of complexes of active 1–325 aa fragment of SC with thrombin and prethrombin-2 revealed that the D1 region of SC contains prothrombin-activating and -binding domains [6]. Therefore, it seems that *S. aureus* strains have changed their antigenecities of SC by altering the amino acids to evade the host immune system. It was reported that pooled normal human gamma-globulin inhibits the clotting activity of staphylocoagulase produced by 24 different staphylococcal bacteriophage propagating strains [47]. Although clotting activities of SCs from 19 strains of them were inhibited for at least 24 hr by pooled normal human gamma-globulin, with the 5 remaining strains, gamma-globulin also inhibited clotting but for a shorter period of time (4–5 hr). The data indicate that humans actually have anti-staphylocoagulase antibodies and they couldn't effectively inhibit the staphylocoagulse activity of all SC types.

It has also been speculated that these variation in D1 regions might be useful to adapt to the different SC-attachment sites in prothrombin among mammalian species [5]. In this study, we analyzed *S. aureus* strains isolated from three different species, human, cow and cat. We also analyzed two strains from humans with infection of isolates of ST398, which have been related to pigs. We could not find the animal species-specific SC types. However, there are some differences of SC types between the host species. We previously reported that 65 SC type VI strains isolated from cow were classified into two subtypes, type VIb and VIc, whereas most of the SC type VI strains isolated from humans were subtyped into VIa [7]. It was also described that a fully active SC partial fragment, SC-(1–325) of SC type I strain 104, activates bovine prothrombin, but the SC-(1–325)·bovine prothrombin complex is about 5,800-fold less active than its human counterpart [48]. Structural analysis showed the less activity is mainly due to structural collision of N-terminal insertion into the bovine (pro) thrombin Ile16 pocket, which is an important step to activate prothrombin. Strain 104 was isolated from human. It is interesting whether type VIb and VIc SC·human prothrombin complex is similarly less active than its bovine counterpart for activation of bovine prothrombin and vice versa. If so this could indicate that SC is host specific. In this study, 29 strains isolated from cats were analyzed by M-PCRs and eighteen *coa* of them were sequenced. Seven of eighteen sequenced strains from cats had different SC subtypes (Ib, IIb, VIIa/b and VIIIb) from those of human isolates. Two *coa* of strains isolated from patients associated with pigs were sequenced and typed SC type VIIb. However, type VIIb strains were also found from isolates of ST45 and ST508, the ST45 strains are well established human pathogens and the ST508 strains were isolated from cat.

Besides *S. aureus*, six species of other coagulase positive staphylococci have been reported, *S. intermedius*, *S. pseudintermedius*, *S. schleiferi* subsp. *coagulans*, *S. hyicus*, *S. lutrae* and *S. delphini* [49]–[51]. Similar to *S. aureus* they colonize the skin and mucosal membrane of warm-blood animals, and more frequently cause animal infections than coagulase-negative staphylococci [52]. It is interesting that coagulase-positive staphylococci tend to have host specificity [52], [53]. We could not detect *coa* in the six species by PCR with primers designed based on the *coa* sequences of *S. aureus* (data not shown). Staphylocoagulase produced by *S. intermedius* is distinct from that of *S. aureus* because coagulase produced by *S. intermedius* is a glycoprotein and the N terminus of coagulase of *S. intermedius* is different from that of *S. aureus*, which is essential for prothrombin activation [54]. Moreover they are different in antigenicity [55]. It is suggested that non-aureus strains have different proteins from that of *S. aureus* but similar activity to clot serum or they have coagulase similar to that of *S. aureus* but the sequences of *coa* are diverged. In turn, possession of different type of coagulase and the presumed diversity of the *coa* sequences in the non-aureus strains could imply that SC plays a role in host specificity. Alternatively, one might expect that orthologous loci in other species could be diverse regardless of their role in host specificity.

Here we showed evidence for the recombination events between different *coa* alleles. The diversification of SC may be a key strategy for *S. aureus* to circumvent humoral immunity elicited by the host immune systems.

## Supporting Information

Table S1 Characteristics of the 126 *S. aureus* strains examined in this study
(0.02 MB PDF)

Table S2 Nucleotide and amino acid identities on D1, D2 and central regions among same SC types Identities of nucleotide sequences of D1, D2 and central regions of *coa* and their deduced amino acid sequences among each same SC type. Nucleotide identities are shown in cells in the bottom left half of the table and amino acid identities are shown in cells in the upper right half of the table.
(0.04 MB PDF)
