# Supplementary material for: Genetic Diversity of Staphylocoagulase Genes (coa): Insight into the Evolution of Variable Chromosomal Virulence Factors in Staphylococcus aureus
Source: PLoS One. 2009 May 27;4(5):e5714. doi: 10.1371/journal.pone.0005714 (PMC2683563; doi:10.1371/journal.pone.0005714)
Supplement: Table S1 — Characteristics of the 126 S. aureus strains examined in this study (0.02 MB PDF) [file pone.0005714.s001.pdf]

TableS1. Characteristics of the 126 *S. aureus* strains examined in this study

	strain name	SC[a]	Sero[b]	*agr*	CC[c]	ST[d]	MR[e]	comment	length of *coa*	repeat[f]	reference	Accession No.
1	104	Ia	I	II	Minor group	ST49	MSSA	SC reffernce strain	2061	7	4	AB158549
2	NVAU02066	Ib	I	I	CC133	ST133	MSSA	isolated from a cat in Japan	2145	8	this study	AB436972
3	NVAU02069	Ib	I	I	CC133	ST133	MSSA	isolated from a cat in Japan	2145	8	this study	AB488502
4	NVAU02072	I	I	I	CC133	ST133	MSSA	isolated from a cat in Japan	Not Seq[k]	Not Seq[k]	this study	Not Seq[k]
5	TSCC10	I	I	I	CC133	ST133	MSSA	isolated from a cat in Japan	Not Seq[k]	Not Seq[k]	this study	Not Seq[k]
6	SD036-1	Ic	I	III	CC91	ST89	MRSA	CA-MRSA isolated in Japan	2037	6	16	AB489888
7	JCSC4762	Ic	I	III	CC91	ST89	MRSA	HA-MRSA isolated in Japan	2037	6	14	AB489876
8	JCSC4766	I	I	III	CC91	ST89	MRSA	HA-MRSA isolated in Japan	Not Seq[k]	Not Seq[k]	14	Not Seq[k]
9	JCSC4770	I	I	III	CC91	ST89	MRSA	HA-MRSA isolated in Japan	Not Seq[k]	Not Seq[k]	14	Not Seq[k]
10	SD2:176-3	Ic	I	III	CC91	ST91	MRSA	CA-MRSA isolated in Japan	2037	6	16	AB489889
11	W12	Ic	I	III	CC91	ST91	MRSA	CA-MRSA isolated in Japan	2037	6	16	AB489890
12	JCSC4752	Ic	I	III	CC91	ST91	MRSA	HA-MRSA isolated in Japan	2037	6	14	AB489875
13	JCSC4765	I	I	III	CC91	ST91	MRSA	HA-MRSA isolated in Japan	Not Seq[k]	Not Seq[k]	14	Not Seq[k]
14	JCSC4769	I	I	III	CC91	ST91	MRSA	HA-MRSA isolated in Japan	Not Seq[k]	Not Seq[k]	14	Not Seq[k]
15	JCSC4774	Ic	I	III	CC91	ST376	MRSA	HA-MRSA isolated in Japan	2037	6	14	AB489877
16	JCSC4795	I	I	III	CC91	ST376	MRSA	HA-MRSA isolated in Japan	Not Seq[k]	Not Seq[k]	14	Not Seq[k]
17	JCSC4744	Ic	I	III	CC91	ST379	MRSA	HA-MRSA isolated in Japan	2037	6	14	AB436973
18	JCSC4784	Ic	I	III	CC91	ST379	MRSA	HA-MRSA isolated in Japan	2037	6	14	AB489878
19	Mu3	IIa	II	II	CC5	ST5	MRSA	whole genome-sequenced strain	1977	6	31	AP009324
20	N315	IIa	II	II	CC5	ST5	MRSA	whole genome-sequenced strain	1977	6	29	BA000018
21	Mu50	IIa	II	II	CC5	ST5	MRSA	whole genome-sequenced strain	1977	6	29	BA000017
22	NVAU02075	IIa	II	II	CC5	ST5	MRSA	isolated from a cat in Japan	1977	6	this study	AB488505
23	JCSC6670	IIa	II	II	CC5	ST5	MRSA	isolated in Sweden	1977	6	15	AB489898
24	AIS2002060 (USA800)	IIa	II	II	CC5	ST5	MRSA	major MRSA clones isolated in USA	1977	6	18	AB489886
25	AIS2002053 (USA100)	IIa	II	II	CC5	ST5	MRSA	major MRSA clones isolated in USA	1977	6	18	AB489881
26	NVAU02067	IIa	II	II	CC5	ST5	MSSA	isolated from a cat in Japan	1977	6	this study	AB488501
27	NVAU02076	II	II	II	CC5	ST5	MRSA	isolated from a cat in Japan	Not Seq[k]	Not Seq[k]	this study	Not Seq[k]
28	NVAU02077	II	II	II	CC5	ST5	MRSA	isolated from a cat in Japan	Not Seq[k]	Not Seq[k]	this study	Not Seq[k]
29	NVAU02078	II	II	II	CC5	ST5	MRSA	isolated from a cat in Japan	Not Seq[k]	Not Seq[k]	this study	Not Seq[k]
30	NVAU02073	II	II	II	CC5	ST5	MSSA	isolated from a cat in Japan	Not Seq[k]	Not Seq[k]	this study	Not Seq[k]
31	NVAU02068	II	II	II	CC5	ST5	MSSA	isolated from a cat in Japan	Not Seq[k]	Not Seq[k]	this study	Not Seq[k]
32	TSCC11	II	II	II	CC5	ST5	MSSA	isolated from a cat in Japan	Not Seq[k]	Not Seq[k]	this study	Not Seq[k]
33	SAP260	IIa	II	II	CC5	ST73	MRSA	CA-MRSA isolated in Australia	1977	6	17	AB489891
34	JH1	IIa	-[i]	II	CC5	ST105	MRSA	whole genome-sequenced strain	1977	6	30	CP000736
35	JH9	IIa	-[i]	II	CC5	ST105	MRSA	whole genome-sequenced strain	1977	6	30	CP000703
36	NVAU02064	IIb	II	I	CC25	ST25	MSSA	isolated from a cat in Japan	1992	5	this study	AB436974
37	NVAU02074	IIb	II	I	CC25	ST25	MSSA	isolated from a cat in Japan	1992	5	this study	AB488504
38	FPR3757 (USA300)	IIIa	III	I	CC8	ST8	MRSA	whole genome-sequenced strain	1830	4	23	CP000255
39	TCH1516 (USA300)	IIIa	-[i]	I	CC8	ST8	MRSA	whole genome-sequenced strain	1830	4	27	CP000730
40	AIS2002055 (USA300)	IIIa	III	I	CC8	ST8	MRSA	major MRSA clones isolated in USA	1830	4	18	AB489883
41	NCTC8325	IIIa	III	I	CC8	ST8	MSSA	whole genome-sequenced strain	1911	5	25	CP000253
42	86/4372	IIIa	III	I	CC8	ST8	MRSA	isolated in UK	1911	5	12	AB436957
43	AIS2002057 (USA500)	IIIa	III	I	CC8	ST8	MRSA	major MRSA clones isolated in USA	1911	5	18	AB489885
44	COL	IIIa	III	I	CC8	ST250	MRSA	whole genome-sequenced strain	1911	5	24	CP000046
45	NCTC10442	IIIa	III	I	CC8	ST250	MRSA	isolated in UK	1911	5	12	AB436955
46	61/6219	IIIa	III	I	CC8	ST250	MRSA	isolated in UK	1911	5	12	AB436956
47	Newman	IIIa	III	I	CC8	ST254	MSSA	whole genome-sequenced strain	1911	5	21	AP009351
48	JCSC6663	IIIa	LP[g]	I	CC8	ST254	MRSA	isolated in Sweden	1911	5	15	AB489897
49	JCSC4741	IIIa	III	I	CC8	ST380	MRSA	HA-MRSA isolated in Japan	1911	5	14	AB489873
50	NVAU02079	III	III	I	CC8	ST380	MRSA	isolated from a cat in Japan	Not Seq[k]	Not Seq[k]	this study	Not Seq[k]
51	JCSC4746	IIIa	III	I	CC8	ST450	MRSA	HA-MRSA isolated in Japan	1818	4	14	AB489874
52	JCSC4788	III	III	I	CC8	ST451	MRSA	HA-MRSA isolated in Japan	Not Seq[k]	Not Seq[k]	14	Not Seq[k]
53	TSCC26	IIIa	III	I	CC8	ST995	MRSA	isolated from a cat in Japan	1587	1	this study	AB488510
54	TSCC12	IIIa	III	I	CC8	ST1252	MSSA	isolated from a cat in Japan	1911	5	this study	AB488500
55	TSCC14	III	III	I	CC8	ST1253	MSSA	isolated from a cat in Japan	Not Seq[k]	Not Seq[k]	this study	Not Seq[k]
56	NVAU02080	IIIa	III	II	Minor group	ST1251	MSSA	isolated from a cat in Japan	2235	9	this study	AB436975
57	NVAU02070	IIIa	III	II	Minor group	ST1251	MSSA	isolated from a cat in Japan	2235	9	this study	AB488499
58	M9N	IIIa	III	III	CC88	ST78	MRSA	CA-MRSA isolated in Australia	2073	7	17	AB436976
59	M33T	III	III	III	CC88	ST78	MRSA	CA-MRSA isolated in Australia	Not Seq[k]	Not Seq[k]	17	Not Seq[k]
60	W17	IIIa	III	III	CC88	ST88	MRSA	CA-MRSA isolated in Japan	2073	7	16	AB489892
61	85/2082	IVa	IV	I	CC8	ST239	MRSA	isolated in New Zealand	1992	6	12	AB436958
62	85/3907	IVa	IV	I	CC8	ST239	MRSA	isolated in Germany	1992	6	12	AB436959
63	86/961	IVa	IV	I	CC8	ST239	MRSA	isolated in UK	1992	6	12	AB436960
64	stp28	IVa	IV	III	CC30	ST30	MSSA	SC reffernce strain	1992	6	4	AB158550
65	93/H44	IVa	IV	III	CC30	ST30	MRSA	isolated in Japan	1992	6	12	AB436961
66	85/2232	IVa	IV	III	CC30	ST30	MRSA	isolated in Japan	1992	6	12	AB436962
67	JCSC6673	IVa	IV	III	CC30	ST30	MRSA	isolated in Sweden	1992	6	15	AB489880
68	AIS2002062 (USA1100)	IVa	IV	III	CC30	ST30	MRSA	major MRSA clones isolated in USA	1992	6	18	AB489887
69	TSCC15	IVa	LP[g]	III	CC30	ST30	MSSA	isolated from a cat in Japan	1992	6	this study	AB488508
70	MRSA252	IVa	IV	III	CC30	ST36	MRSA	whole genome-sequenced strain	1830	4	28	BX571856
71	AIS2002054 (USA200)	IVa	IV	III	CC30	ST36	MRSA	major MRSA clones isolated in USA	1830	4	18	AB489882
72	MR108	IVa	IV	III	CC30	ST74	MRSA	isolated in Japan	1992	6	13	AB436963
73	SH488	IVb	NT[h]	I	CC6	ST6	MSSA	isolated in Japan	Partial seq[l]	Partial seq[l]	20	from Dr Kobayashi

74	No55	Va	V	IV	CC51	ST95	MSSA	SC reffernce strain	2178	8	4	AB158551
75	Stp-58	Va	V	IV	CC51	ST120	MSSA	isolated in Japan	2178	8	this study	AB489894
76	Stp-24	V	V	IV	CC51	ST120	MSSA	isolated in Japan	Not Seq[k]	Not Seq[k]	this study	Not Seq[k]
77	Stp-25	Va	V	IV	CC51	ST121	MSSA	isolated in Japan	2178	8	this study	AB489895
78	01093	Vb	V	I	CC8	ST72	MRSA	CA-MRSA isolated in USA	1962	6	17	AB436978
79	AIS2002059 (USA700)	Vb	V	I	CC8	ST72	MRSA	major MRSA clones isolated in USA	1962	6	18	AB436979
80	C-1C	Vb	NT[h]	I	CC1	ST188	MSSA	isolated from a cat in Japan	1962	6	this study	AB436977
81	TSCC17	Vb	V	I	CC1	ST188	MSSA	isolated from a cat in Japan	1962	6	this study	AB488509
82	SH640	Vb	NT[h]	I	CC1	ST188	MSSA	isolated in Japan	Partial seq[l]	Partial seq[l]	20	from Dr Kobayashi
83	stp12	VIa	VI	III	Minor group	ST96	MSSA	SC reffernce strain	1989	6	4	AB158552
84	RF122 (ET3-1)	VIb	VI	II	CC151	ST151	MSSA	whole genome-sequenced strain	1884	5	26	AJ938182
85	IFH556	VIb	VI	II	CC151	ST705	MSSA	isolated from a cow in Japan	1884	5	7	AB373752
86	IFH812	VIb	VI	II	CC151	ST705	MSSA	isolated from a cow in Japan	1884	5	7	AB373753
87	IFH818	VIb	VI	II	CC151	ST705	MSSA	isolated from a cow in Japan	1884	5	7	AB373754
88	strain M (JCSC6488)	VIc	LP[g]	II	Singleton	ST1254	MSSA	type I capsule-producing strain	1404	4	11	AB436980
89	IFH514	VIc	VI	I	CC97	ST352	MSSA	isolated from a cow in Japan	2151	8	7	AB373755
90	IFH568	VIc	VI	I	CC97	ST352	MSSA	isolated from a cow in Japan	2151	8	7	AB373756
91	IFH467	VIc	VI	I	CC97	ST352	MSSA	isolated from a cow in Japan	2151	8	7	AB373757
92	MSSA476	VIIa	VII	III	CC1	ST1	MSSA	whole genome-sequenced strain	1902	5	28	BX571857
93	MW2	VIIa	VII	III	CC1	ST1	MRSA	whole genome-sequenced strain	1902	5	22	BA000033
94	JCSC6076	VIIa	VII	III	CC1	ST1	MRSA	isolated in Sweden	1902	5	15	AB489899
95	AIS2002056 (USA400)	VIIa	VII	III	CC1	ST1	MRSA	major MRSA clones isolated in USA	1902	5	18	AB489884
96	JCSC6668	VII	VII	III	CC1	ST1	MRSA	isolated in Sweden	Not Seq[k]	Not Seq[k]	15	Not Seq[k]
97	91/2619	VIIa	VII	III	CC1	ST76	MRSA	CA-MRSA isolated in Australia	1902	5	17	AB489901
98	JCSC4796	VIIa	VII	III	CC1	ST81	MRSA	HA-MRSA isolated in Japan	1902	5	14	AB489879
99	TSCC9A	VIIa	LP[g]	III	CC1	ST81	MSSA	isolated from a cat in Japan	1902	5	this study	AB488507
100	TSCC9B	VII	LP[g]	III	CC1	ST81	MSSA	isolated from a cat in Japan	Not Seq[k]	Not Seq[k]	this study	Not Seq[k]
101	NVAU02063	VIIa/b	VII	I	Singleton	ST1253	MSSA	isolated from a cat in Japan	2145	8	this study	AB488498
102	JCSC6665	VIIb	NT[h]	I	CC45	ST45	MRSA	isolated in Sweden	2016	6	15	AB436964
103	JCSC6068	VIIb	VII	I	CC45	ST45	MRSA	isolated in Sweden	2016	6	15	AB489900
104	WIS	VIIb	VII	I	CC45	ST45	MRSA	CA-MRSA isolated in Australia	2016	6	17	AB489893
105	AIS2002058 (USA600)	VIIb	LP[g]	I	CC45	ST45	MRSA	major MRSA clones isolated in USA	1773	3	18	AB436981
106	JCSC6073	VII	VII	I	CC45	ST45	MRSA	isolated in Sweden	Not Seq[k]	Not Seq[k]	15	Not Seq[k]
107	C-12B	VIIb	NT[h]	I	CC45	ST508	MSSA	isolated from a cat in Japan	2016	6	this study	AB488506
108	JCSC6908	VIIb	VII	I	CC398	ST398	MRSA	isolated in Denmark	2097	7	19	AB436983
109	JCSC6909	VIIb	NT[h]	I	CC398	ST398	MRSA	isolated in Denmark	2097	7	19	AB436984
110	AIS2002061 (USA1000)	VIIc	LP[g]	I	CC59	ST59	MRSA	major MRSA clones isolated in USA	2064	7	18	AB436982
111	JCSC6664	VIIc	NT[h]	I	CC59	ST59	MRSA	isolated in Sweden	2064	7	15	AB489896
112	Ku	VIIIa	VIII	ND[j]	Minor group	ST10	MSSA	SC reffernce strain	2280	9	4	AB158553
113	NVAU02081	VIIIb	VIII	I	CC20	ST20	MSSA	isolated from a cat in Japan	1935	5	this study	AB436985
114	NVAU02071	VIIIb	VIII	I	CC20	ST20	MSSA	isolated from a cat in Japan	1935	5	this study	AB488503
115	17573	IXa	IX	III	CC130	ST574	MSSA	SC reffernce strain	1992	6	3	AB158554
116	19	Xa	X	II	CC15	ST15	MSSA	SC reffernce strain	1935	5	3	AB158555
117	JCSC6074	Xb	LP[g]	IV	Minor group	ST140	MRSA	isolated in Sweden	1983	6	15	AB436965
118	JCSC6666	Xb	LP[g]	IV	Minor group	ST140	MRSA	isolated in Sweden	1983	6	15	AB436966
119	JCSC6075	XIa	LP[g]	I	CC22	ST22	MRSA	isolated in Sweden	1971	6	15	AB436967
120	JCSC6667	XIa	LP[g]	I	CC22	ST22	MRSA	isolated in Sweden	1971	6	15	AB436968
121	JCSC6671	XIa	LP[g]	I	CC22	ST22	MRSA	isolated in Sweden	1971	6	15	AB436969
122	JCSC6674	XIa	LP[g]	I	CC22	ST22	MRSA	isolated in Sweden	1971	6	15	AB436970
123	JCSC6906	XIa	LP[g]	I	CC22	ST22	MRSA	isolated in Denmark	1971	6	this study	AB436986
124	JCSC6907	XIa	LP[g]	I	CC22	ST22	MRSA	isolated in Denmark	1971	6	this study	AB436987
125	JCSC6669	XIb	LP[g]	I	CC182	ST182	MRSA	isolated in Sweden	2037	6	15	AB436971
126	JCSC1469 (SAP411)	XIIa	VI	I	Minor group	ST75	MRSA	CA-MRSA isolated in Australia	1974	6	17	AB436988

[a] Staphylocoagulase type. SC subtypes are indicated only in the cases of the *coa* -sequenced strains. SCs of the other strains, of which SC types were determined with M-PCR, were not subtyped.

[b] Staphylocoagulase serotype [c] Clonal complex, [d] Sequence type of MLST, [e] Methicilin resistance, [f] number of repeat units in *coa* ,

[g] Staphylocoagulase production was too low to clot serum within 48 hr, [h] Non typable, [i] Not tested, [j] Not detected by M-PCR for *agr* , [k] Not sequenced, [l] Partially sequenced
