# Supplementary material for: Genetic Diversity of Staphylocoagulase Genes (coa): Insight into the Evolution of Variable Chromosomal Virulence Factors in Staphylococcus aureus
Source: PLoS One. 2009 May 27;4(5):e5714. doi: 10.1371/journal.pone.0005714 (PMC2683563; doi:10.1371/journal.pone.0005714)
Supplement: Table S2 — Nucleotide and amino acid identities on D1, D2 and central regions among same SC types Identities of nucleotide sequences of D1, D2 and central regions of coa and their deduced amino acid sequences among each same SC type. Nucleotide identities are shown in cells in the bottom left half of the table and amino acid identities are shown in cells in the upper right half of the table. (0.04 MB PDF) [file pone.0005714.s002.pdf]

Supplemetal data

TableS2. Nucleotide and amino acid identities on D1, D2 and central regions among same SC types

Identities of nucleotide sequences of D1, D2 and central regions of coa and their deduced amino acid sequences among each same SC type. Nucleotide identities are shown in cells in the bottom left half of the table and amino acid identities are shown in cells in the upper right half of the table.

S2-1. SC type I

a. D1 region

strain name	CC	ST	*agr*	SC	104	NVAU02066	NVAU02069	SD036-1	JCSC4762	SD2:176-3	W12	JCSC4752	JCSC4774	JCSC4744	JCSC4784
104	Minor group	ST49	II	Ia		98.7%	98.7%	97.3%	97.3%	97.3%	96.0%	97.3%	97.3%	97.3%	97.3%
NVAU02066	CC133	ST133	I	Ib	99.1%		100.0%	98.0%	98.0%	98.0%	96.6%	98.0%	98.0%	98.0%	98.0%
NVAU02069	CC133	ST133	I	Ib	99.1%	100.0%		98.0%	98.0%	98.0%	96.6%	98.0%	98.0%	98.0%	98.0%
SD036-1	CC91	ST89	III	Ic	97.8%	98.0%	98.0%		100.0%	100.0%	98.7%	100.0%	100.0%	100.0%	100.0%
JCSC4762	CC91	ST89	III	Ic	97.8%	98.0%	98.0%	100.0%		100.0%	98.7%	100.0%	100.0%	100.0%	100.0%
SD2:176-3	CC91	ST91	III	Ic	97.8%	98.0%	98.0%	100.0%	100.0%		98.7%	100.0%	100.0%	100.0%	100.0%
W12	CC91	ST91	III	Ic	97.3%	97.5%	97.5%	99.6%	99.6%	99.6%		98.7%	98.7%	98.7%	98.7%
JCSC4752	CC91	ST91	III	Ic	97.8%	98.0%	98.0%	100.0%	100.0%	100.0%	99.6%		100.0%	100.0%	100.0%
JCSC4774	CC91	ST376	III	Ic	97.8%	98.0%	98.0%	100.0%	100.0%	100.0%	99.6%	100.0%		100.0%	100.0%
JCSC4744	CC91	ST379	III	Ic	97.8%	98.0%	98.0%	100.0%	100.0%	100.0%	99.6%	100.0%	100.0%		100.0%
JCSC4784	CC91	ST379	III	Ic	97.8%	98.0%	98.0%	100.0%	100.0%	100.0%	99.6%	100.0%	100.0%	100.0%	

b. D2 region

strain name	CC	ST	*agr*	SC	104	NVAU02066	NVAU02069	SD036-1	JCSC4762	SD2:176-3	W12	JCSC4752	JCSC4774	JCSC4744	JCSC4784
104	Minor group	ST49	II	Ia		96.2%	96.2%	98.5%	98.5%	98.5%	97.7%	98.5%	98.5%	98.5%	98.5%
NVAU02066	CC133	ST133	I	Ib	97.4%		100.0%	96.9%	96.9%	96.9%	96.2%	96.9%	96.9%	96.9%	96.9%
NVAU02069	CC133	ST133	I	Ib	97.4%	100.0%		96.9%	96.9%	96.9%	96.2%	96.9%	96.9%	96.9%	96.9%
SD036-1	CC91	ST89	III	Ic	99.5%	97.7%	97.7%		100.0%	100.0%	99.2%	100.0%	100.0%	100.0%	100.0%
JCSC4762	CC91	ST89	III	Ic	99.5%	97.7%	97.7%	100.0%		100.0%	99.2%	100.0%	100.0%	100.0%	100.0%
SD2:176-3	CC91	ST91	III	Ic	99.5%	97.7%	97.7%	100.0%	100.0%		99.2%	100.0%	100.0%	100.0%	100.0%
W12	CC91	ST91	III	Ic	99.2%	97.4%	97.4%	99.7%	99.7%	99.7%		99.2%	99.2%	99.2%	99.2%
JCSC4752	CC91	ST91	III	Ic	99.5%	97.7%	97.7%	100.0%	100.0%	100.0%	99.7%		100.0%	100.0%	100.0%
JCSC4774	CC91	ST376	III	Ic	99.5%	97.7%	97.7%	100.0%	100.0%	100.0%	99.7%	100.0%		100.0%	100.0%
JCSC4744	CC91	ST379	III	Ic	99.5%	97.7%	97.7%	100.0%	100.0%	100.0%	99.7%	100.0%	100.0%		100.0%
JCSC4784	CC91	ST379	III	Ic	99.5%	97.7%	97.7%	100.0%	100.0%	100.0%	99.7%	100.0%	100.0%	100.0%	

c. Central region

strain name	CC	ST	*agr*	SC	104	NVAU02066	NVAU02069	SD036-1	JCSC4762	SD2:176-3	W12	JCSC4752	JCSC4774	JCSC4744	JCSC4784
104	Minor group	ST49	II	Ia		84.0%	84.0%	77.7%	77.7%	77.7%	77.7%	77.7%	77.7%	77.7%	77.7%
NVAU02066	CC133	ST133	I	Ib	89.7%		100.0%	85.4%	85.4%	85.4%	85.4%	85.4%	85.4%	85.4%	85.4%
NVAU02069	CC133	ST133	I	Ib	89.7%	100.0%		85.4%	85.4%	85.4%	85.4%	85.4%	85.4%	85.4%	85.4%
SD036-1	CC91	ST89	III	Ic	81.6%	85.0%	85.0%		100.0%	100.0%	99.5%	100.0%	100.0%	100.0%	100.0%
JCSC4762	CC91	ST89	III	Ic	81.6%	85.0%	85.0%	100.0%		100.0%	99.5%	100.0%	100.0%	100.0%	100.0%
SD2:176-3	CC91	ST91	III	Ic	81.6%	85.0%	85.0%	100.0%	100.0%		99.5%	100.0%	100.0%	100.0%	100.0%
W12	CC91	ST91	III	Ic	81.7%	86.6%	86.6%	99.8%	99.8%	99.8%		99.5%	99.5%	99.5%	99.5%
JCSC4752	CC91	ST91	III	Ic	81.6%	85.0%	85.0%	100.0%	100.0%	100.0%	99.8%		100.0%	100.0%	100.0%
JCSC4774	CC91	ST376	III	Ic	81.6%	85.0%	85.0%	100.0%	100.0%	100.0%	99.8%	100.0%		100.0%	100.0%
JCSC4744	CC91	ST379	III	Ic	81.6%	85.0%	85.0%	100.0%	100.0%	100.0%	99.8%	100.0%	100.0%		100.0%
JCSC4784	CC91	ST379	III	Ic	81.6%	85.0%	85.0%	100.0%	100.0%	100.0%	99.8%	100.0%	100.0%	100.0%	

S2-2. SC type II

a. D1 region

strain name	CC	ST	agr	SC	Mu50	N315	JH1	JH9	Mu3	NVAU02075	JCSC6670	AIS2002060	AIS2002053	NVAU02067	SAP260	NVAU02064	NVAU02074
Mu50	CC5	ST5	II	IIa		100.0%	100.0%	100.0%	100.0%	100.0%	100.0%	100.0%	100.0%	100.0%	100.0%	99.3%	99.3%
N315	CC5	ST5	II	IIa	100.0%		100.0%	100.0%	100.0%	100.0%	100.0%	100.0%	100.0%	100.0%	100.0%	99.3%	99.3%
Mu3	CC5	ST5	II	IIa	100.0%	100.0%		100.0%	100.0%	100.0%	100.0%	100.0%	100.0%	100.0%	100.0%	99.3%	99.3%
NVAU02075	CC5	ST5	II	IIa	100.0%	100.0%	100.0%		100.0%	100.0%	100.0%	100.0%	100.0%	100.0%	100.0%	99.3%	99.3%
JCSC6670	CC5	ST5	II	IIa	100.0%	100.0%	100.0%	100.0%		100.0%	100.0%	100.0%	100.0%	100.0%	100.0%	99.3%	99.3%
AIS2002060	CC5	ST5	II	IIa	100.0%	100.0%	100.0%	100.0%	100.0%		100.0%	100.0%	100.0%	100.0%	100.0%	99.3%	99.3%
AIS2002053	CC5	ST5	II	IIa	100.0%	100.0%	100.0%	100.0%	100.0%	100.0%		100.0%	100.0%	100.0%	100.0%	99.3%	99.3%
NVAU02067	CC5	ST5	II	IIa	100.0%	100.0%	100.0%	100.0%	100.0%	100.0%	100.0%		100.0%	100.0%	100.0%	99.3%	99.3%
SAP260	CC5	ST73	II	IIa	100.0%	100.0%	100.0%	100.0%	100.0%	100.0%	100.0%	100.0%		100.0%	100.0%	99.3%	99.3%
JH1	CC5	ST105	II	IIa	100.0%	100.0%	100.0%	100.0%	100.0%	100.0%	100.0%	100.0%	100.0%		100.0%	99.3%	99.3%
JH9	CC5	ST105	II	IIa	100.0%	100.0%	100.0%	100.0%	100.0%	100.0%	100.0%	100.0%	100.0%	100.0%		99.3%	99.3%
NVAU02064	CC25	ST25	I	IIb	99.8%	99.8%	99.8%	99.8%	99.8%	99.8%	99.8%	99.8%	99.8%	99.8%	99.8%		100.0%
NVAU02074	CC25	ST25	I	IIb	99.8%	99.8%	99.8%	99.8%	99.8%	99.8%	99.8%	99.8%	99.8%	99.8%	99.8%	100.0%	

b. D2 region

strain name	CC	ST	agr	SC	Mu50	N315	JH1	JH9	Mu3	NVAU02075	JCSC6670	AIS2002060	AIS2002053	NVAU02067	SAP260	NVAU02064	NVAU02074
Mu50	CC5	ST5	II	IIa		100.0%	100.0%	100.0%	100.0%	100.0%	100.0%	100.0%	100.0%	100.0%	100.0%	100.0%	100.0%
N315	CC5	ST5	II	IIa	100.0%		100.0%	100.0%	100.0%	100.0%	100.0%	100.0%	100.0%	100.0%	100.0%	100.0%	100.0%
Mu3	CC5	ST5	II	IIa	100.0%	100.0%		100.0%	100.0%	100.0%	100.0%	100.0%	100.0%	100.0%	100.0%	100.0%	100.0%
NVAU02075	CC5	ST5	II	IIa	100.0%	100.0%	100.0%		100.0%	100.0%	100.0%	100.0%	100.0%	100.0%	100.0%	100.0%	100.0%
JCSC6670	CC5	ST5	II	IIa	100.0%	100.0%	100.0%	100.0%		100.0%	100.0%	100.0%	100.0%	100.0%	100.0%	100.0%	100.0%
AIS2002060	CC5	ST5	II	IIa	100.0%	100.0%	100.0%	100.0%	100.0%		100.0%	100.0%	100.0%	100.0%	100.0%	100.0%	100.0%
AIS2002053	CC5	ST5	II	IIa	100.0%	100.0%	100.0%	100.0%	100.0%	100.0%		100.0%	100.0%	100.0%	100.0%	100.0%	100.0%
NVAU02067	CC5	ST5	II	IIa	100.0%	100.0%	100.0%	100.0%	100.0%	100.0%	100.0%		100.0%	100.0%	100.0%	100.0%	100.0%
SAP260	CC5	ST73	II	IIa	100.0%	100.0%	100.0%	100.0%	100.0%	100.0%	100.0%	100.0%		100.0%	100.0%	100.0%	100.0%
JH1	CC5	ST105	II	IIa	100.0%	100.0%	100.0%	100.0%	100.0%	100.0%	100.0%	100.0%	100.0%		100.0%	100.0%	100.0%
JH9	CC5	ST105	II	IIa	100.0%	100.0%	100.0%	100.0%	100.0%	100.0%	100.0%	100.0%	100.0%	100.0%		100.0%	100.0%
NVAU02064	CC25	ST25	I	IIb	100.0%	100.0%	100.0%	100.0%	100.0%	100.0%	100.0%	100.0%	100.0%	100.0%	100.0%		100.0%
NVAU02074	CC25	ST25	I	IIb	100.0%	100.0%	100.0%	100.0%	100.0%	100.0%	100.0%	100.0%	100.0%	100.0%	100.0%	100.0%	

c. Central region

strain name	CC	ST	agr	SC	Mu50	N315	Mu3	NVAU02075	JCSC6670	AIS2002060	AIS2002053	NVAU02067	SAP260	JH1	JH9	NVAU02064	NVAU02074
Mu50	CC5	ST5	II	IIa		100.0%	100.0%	100.0%	99.5%	100.0%	100.0%	100.0%	100.0%	100.0%	100.0%	82.3%	82.3%
N315	CC5	ST5	II	IIa	100.0%		100.0%	100.0%	99.5%	100.0%	100.0%	100.0%	100.0%	100.0%	100.0%	82.3%	82.3%
Mu3	CC5	ST5	II	IIa	100.0%	100.0%		100.0%	99.5%	100.0%	100.0%	100.0%	100.0%	100.0%	100.0%	82.3%	82.3%
NVAU02075	CC5	ST5	II	IIa	100.0%	100.0%	100.0%		99.5%	100.0%	100.0%	100.0%	100.0%	100.0%	100.0%	82.3%	82.3%
JCSC6670	CC5	ST5	II	IIa	99.8%	99.8%	99.8%	99.8%		99.5%	99.5%	99.5%	99.5%	99.5%	99.5%	81.8%	81.8%
AIS2002060	CC5	ST5	II	IIa	100.0%	100.0%	100.0%	100.0%	99.8%		100.0%	100.0%	100.0%	100.0%	100.0%	82.3%	82.3%
AIS2002053	CC5	ST5	II	IIa	100.0%	100.0%	100.0%	100.0%	99.8%	100.0%		100.0%	100.0%	100.0%	100.0%	82.3%	82.3%
NVAU02067	CC5	ST5	II	IIa	100.0%	100.0%	100.0%	100.0%	99.8%	100.0%	100.0%		100.0%	100.0%	100.0%	82.3%	82.3%
SAP260	CC5	ST73	II	IIa	100.0%	100.0%	100.0%	100.0%	99.8%	100.0%	100.0%	100.0%		100.0%	100.0%	82.3%	82.3%
JH1	CC5	ST105	II	IIa	100.0%	100.0%	100.0%	100.0%	99.8%	100.0%	100.0%	100.0%	100.0%		100.0%	82.3%	82.3%
JH9	CC5	ST105	II	IIa	100.0%	100.0%	100.0%	100.0%	99.8%	100.0%	100.0%	100.0%	100.0%	100.0%		82.3%	82.3%
NVAU02064	CC25	ST25	I	IIb	81.4%	81.4%	81.4%	81.4%	81.4%	81.4%	81.4%	81.4%	81.4%	81.4%	81.4%		100.0%
NVAU02074	CC25	ST25	I	IIb	81.4%	81.4%	81.4%	81.4%	81.4%	81.4%	81.4%	81.4%	81.4%	81.4%	81.4%	100.0%	

S2-3. SC type III

a. D1 region

strain name	CC	ST	agr	SC	FPR3757	TCH1516	86/4372	NCTC8325	AIS2002055	AIS2002057	61/6219	COL	NCTC10442	Newman	JCSC6663	JCSC4741	JCSC4746	TSCC26	TSCC12	M9N	W17	NVAU02070	NVAU02080
FPR3757	CC8	ST8	I	IIIa		100.0%	100.0%	100.0%	100.0%	100.0%	100.0%	100.0%	100.0%	100.0%	100.0%	100.0%	100.0%	100.0%	100.0%	100.0%	100.0%	98.7%	98.7%
TCH1516	CC8	ST8	I	IIIa	100.0%		100.0%	100.0%	100.0%	100.0%	100.0%	100.0%	100.0%	100.0%	100.0%	100.0%	100.0%	100.0%	100.0%	100.0%	100.0%	98.7%	98.7%
86/4372	CC8	ST8	I	IIIa	100.0%	100.0%		100.0%	100.0%	100.0%	100.0%	100.0%	100.0%	100.0%	100.0%	100.0%	100.0%	100.0%	100.0%	100.0%	100.0%	98.7%	98.7%
NCTC8325	CC8	ST8	I	IIIa	100.0%	100.0%	100.0%		100.0%	100.0%	100.0%	100.0%	100.0%	100.0%	100.0%	100.0%	100.0%	100.0%	100.0%	100.0%	100.0%	98.7%	98.7%
AIS2002055	CC8	ST8	I	IIIa	100.0%	100.0%	100.0%	100.0%		100.0%	100.0%	100.0%	100.0%	100.0%	100.0%	100.0%	100.0%	100.0%	100.0%	100.0%	100.0%	98.7%	98.7%
AIS2002057	CC8	ST8	I	IIIa	100.0%	100.0%	100.0%	100.0%	100.0%		100.0%	100.0%	100.0%	100.0%	100.0%	100.0%	100.0%	100.0%	100.0%	100.0%	100.0%	98.7%	98.7%
61/6219	CC8	ST250	I	IIIa	100.0%	100.0%	100.0%	100.0%	100.0%	100.0%		100.0%	100.0%	100.0%	100.0%	100.0%	100.0%	100.0%	100.0%	100.0%	100.0%	98.7%	98.7%
COL	CC8	ST250	I	IIIa	100.0%	100.0%	100.0%	100.0%	100.0%	100.0%	100.0%		100.0%	100.0%	100.0%	100.0%	100.0%	100.0%	100.0%	100.0%	100.0%	98.7%	98.7%
NCTC10442	CC8	ST250	I	IIIa	100.0%	100.0%	100.0%	100.0%	100.0%	100.0%	100.0%	100.0%		100.0%	100.0%	100.0%	100.0%	100.0%	100.0%	100.0%	100.0%	98.7%	98.7%
Newman	CC8	ST254	I	IIIa	100.0%	100.0%	100.0%	100.0%	100.0%	100.0%	100.0%	100.0%	100.0%		100.0%	100.0%	100.0%	100.0%	100.0%	100.0%	100.0%	98.7%	98.7%
JCSC6663	CC8	ST254	I	IIIa	100.0%	100.0%	100.0%	100.0%	100.0%	100.0%	100.0%	100.0%	100.0%	100.0%		100.0%	100.0%	100.0%	100.0%	100.0%	100.0%	98.7%	98.7%
JCSC4741	CC8	ST380	I	IIIa	100.0%	100.0%	100.0%	100.0%	100.0%	100.0%	100.0%	100.0%	100.0%	100.0%	100.0%		100.0%	100.0%	100.0%	100.0%	100.0%	98.7%	98.7%
JCSC4746	CC8	ST450	I	IIIa	100.0%	100.0%	100.0%	100.0%	100.0%	100.0%	100.0%	100.0%	100.0%	100.0%	100.0%	100.0%		100.0%	100.0%	100.0%	100.0%	98.7%	98.7%
TSCC26	CC8	ST995	I	IIIa	100.0%	100.0%	100.0%	100.0%	100.0%	100.0%	100.0%	100.0%	100.0%	100.0%	100.0%	100.0%	100.0%		100.0%	100.0%	100.0%	98.7%	98.7%
TSCC12	CC8	ST1252	I	IIIa	100.0%	100.0%	100.0%	100.0%	100.0%	100.0%	100.0%	100.0%	100.0%	100.0%	100.0%	100.0%	100.0%	100.0%		100.0%	100.0%	98.7%	98.7%
M9N	CC88	ST78	III	IIIa	100.0%	100.0%	100.0%	100.0%	100.0%	100.0%	100.0%	100.0%	100.0%	100.0%	100.0%	100.0%	100.0%	100.0%	100.0%		100.0%	98.7%	98.7%
W17	CC88	ST88	III	IIIa	100.0%	100.0%	100.0%	100.0%	100.0%	100.0%	100.0%	100.0%	100.0%	100.0%	100.0%	100.0%	100.0%	100.0%	100.0%	100.0%		98.7%	98.7%
NVAU02070	Minor group	ST1251	II	IIIa	99.6%	99.6%	99.6%	99.6%	99.6%	99.6%	99.6%	99.6%	99.6%	99.6%	99.6%	99.6%	99.6%	99.6%	99.6%	99.6%	99.6%		100.0%
NVAU02080	Minor group	ST1251	II	IIIa	99.6%	99.6%	99.6%	99.6%	99.6%	99.6%	99.6%	99.6%	99.6%	99.6%	99.6%	99.6%	99.6%	99.6%	99.6%	99.6%	99.6%	100.0%	

b. D2 region

strain name	CC	ST	agr	SC	FPR3757	TCH1516	86/4372	NCTC8325	AIS2002055	AIS2002057	61/6219	COL	NCTC10442	Newman	JCSC6663	JCSC4741	JCSC4746	TSCC26	TSCC12	M9N	W17	NVAU02070	NVAU02080
FPR3757	CC8	ST8	I	IIIa		100.0%	100.0%	100.0%	100.0%	100.0%	100.0%	100.0%	100.0%	100.0%	100.0%	100.0%	100.0%	100.0%	100.0%	100.0%	100.0%	93.2%	93.2%
TCH1516	CC8	ST8	I	IIIa	100.0%		100.0%	100.0%	100.0%	100.0%	100.0%	100.0%	100.0%	100.0%	100.0%	100.0%	100.0%	100.0%	100.0%	100.0%	100.0%	93.2%	93.2%
86/4372	CC8	ST8	I	IIIa	100.0%	100.0%		100.0%	100.0%	100.0%	100.0%	100.0%	100.0%	100.0%	100.0%	100.0%	100.0%	100.0%	100.0%	100.0%	100.0%	93.2%	93.2%
NCTC8325	CC8	ST8	I	IIIa	100.0%	100.0%	100.0%		100.0%	100.0%	100.0%	100.0%	100.0%	100.0%	100.0%	100.0%	100.0%	100.0%	100.0%	100.0%	100.0%	93.2%	93.2%
AIS2002055	CC8	ST8	I	IIIa	100.0%	100.0%	100.0%	100.0%		100.0%	100.0%	100.0%	100.0%	100.0%	100.0%	100.0%	100.0%	100.0%	100.0%	100.0%	100.0%	93.2%	93.2%
AIS2002057	CC8	ST8	I	IIIa	100.0%	100.0%	100.0%	100.0%	100.0%		100.0%	100.0%	100.0%	100.0%	100.0%	100.0%	100.0%	100.0%	100.0%	100.0%	100.0%	93.2%	93.2%
61/6219	CC8	ST250	I	IIIa	100.0%	100.0%	100.0%	100.0%	100.0%	100.0%		100.0%	100.0%	100.0%	100.0%	100.0%	100.0%	100.0%	100.0%	100.0%	100.0%	93.2%	93.2%
COL	CC8	ST250	I	IIIa	100.0%	100.0%	100.0%	100.0%	100.0%	100.0%	100.0%		100.0%	100.0%	100.0%	100.0%	100.0%	100.0%	100.0%	100.0%	100.0%	93.2%	93.2%
NCTC10442	CC8	ST250	I	IIIa	100.0%	100.0%	100.0%	100.0%	100.0%	100.0%	100.0%	100.0%		100.0%	100.0%	100.0%	100.0%	100.0%	100.0%	100.0%	100.0%	93.2%	93.2%
Newman	CC8	ST254	I	IIIa	100.0%	100.0%	100.0%	100.0%	100.0%	100.0%	100.0%	100.0%	100.0%		100.0%	100.0%	100.0%	100.0%	100.0%	100.0%	100.0%	93.2%	93.2%
JCSC6663	CC8	ST254	I	IIIa	100.0%	100.0%	100.0%	100.0%	100.0%	100.0%	100.0%	100.0%	100.0%	100.0%		100.0%	100.0%	100.0%	100.0%	100.0%	100.0%	93.2%	93.2%
JCSC4741	CC8	ST380	I	IIIa	100.0%	100.0%	100.0%	100.0%	100.0%	100.0%	100.0%	100.0%	100.0%	100.0%	100.0%		100.0%	100.0%	100.0%	100.0%	100.0%	93.2%	93.2%
JCSC4746	CC8	ST450	I	IIIa	100.0%	100.0%	100.0%	100.0%	100.0%	100.0%	100.0%	100.0%	100.0%	100.0%	100.0%	100.0%		100.0%	100.0%	100.0%	100.0%	93.2%	93.2%
TSCC26	CC8	ST995	I	IIIa	100.0%	100.0%	100.0%	100.0%	100.0%	100.0%	100.0%	100.0%	100.0%	100.0%	100.0%	100.0%	100.0%		100.0%	100.0%	100.0%	93.2%	93.2%
TSCC12	CC8	ST1252	I	IIIa	100.0%	100.0%	100.0%	100.0%	100.0%	100.0%	100.0%	100.0%	100.0%	100.0%	100.0%	100.0%	100.0%	100.0%		100.0%	100.0%	93.2%	93.2%
M9N	CC88	ST78	III	IIIa	100.0%	100.0%	100.0%	100.0%	100.0%	100.0%	100.0%	100.0%	100.0%	100.0%	100.0%	100.0%	100.0%	100.0%	100.0%		100.0%	93.2%	93.2%
W17	CC88	ST88	III	IIIa	100.0%	100.0%	100.0%	100.0%	100.0%	100.0%	100.0%	100.0%	100.0%	100.0%	100.0%	100.0%	100.0%	100.0%	100.0%	100.0%		93.2%	93.2%
NVAU02070	Minor group	ST1251	II	IIIa	95.0%	95.0%	95.0%	95.0%	95.0%	95.0%	95.0%	95.0%	95.0%	95.0%	95.0%	95.0%	95.0%	95.0%	95.0%	95.0%	95.0%		100.0%
NVAU02080	Minor group	ST1251	II	IIIa	95.0%	95.0%	95.0%	95.0%	95.0%	95.0%	95.0%	95.0%	95.0%	95.0%	95.0%	95.0%	95.0%	95.0%	95.0%	95.0%	95.0%	100.0%	

c. Central region

strain name	CC	ST	agr	SC	FPR3757	TCH1516	86/4372	NCTC8325	AIS2002055	AIS2002057	61/6219	COL	NCTC10442	Newman	JCSC6663	JCSC4741	JCSC4746	TSCC26	TSCC12	M9N	W17	NVAU02070	NVAU02080
FPR3757	CC8	ST8	I	IIIa		100.0%	100.0%	100.0%	100.0%	100.0%	100.0%	100.0%	100.0%	100.0%	100.0%	100.0%	100.0%	100.0%	100.0%	96.8%	96.8%	86.7%	86.7%
TCH1516	CC8	ST8	I	IIIa	100.0%		100.0%	100.0%	100.0%	100.0%	100.0%	100.0%	100.0%	100.0%	100.0%	100.0%	100.0%	100.0%	100.0%	96.8%	96.8%	86.7%	86.7%
86/4372	CC8	ST8	I	IIIa	100.0%	100.0%		100.0%	100.0%	100.0%	100.0%	100.0%	100.0%	100.0%	100.0%	100.0%	100.0%	100.0%	100.0%	96.8%	96.8%	86.7%	86.7%
NCTC8325	CC8	ST8	I	IIIa	100.0%	100.0%	100.0%		100.0%	100.0%	100.0%	100.0%	100.0%	100.0%	100.0%	100.0%	100.0%	100.0%	100.0%	96.8%	96.8%	86.7%	86.7%
AIS2002055	CC8	ST8	I	IIIa	100.0%	100.0%	100.0%	100.0%		100.0%	100.0%	100.0%	100.0%	100.0%	100.0%	100.0%	100.0%	100.0%	100.0%	96.8%	96.8%	86.7%	86.7%
AIS2002057	CC8	ST8	I	IIIa	100.0%	100.0%	100.0%	100.0%	100.0%		100.0%	100.0%	100.0%	100.0%	100.0%	100.0%	100.0%	100.0%	100.0%	96.8%	96.8%	86.7%	86.7%
61/6219	CC8	ST250	I	IIIa	100.0%	100.0%	100.0%	100.0%	100.0%	100.0%		100.0%	100.0%	100.0%	100.0%	100.0%	100.0%	100.0%	100.0%	96.8%	96.8%	86.7%	86.7%
COL	CC8	ST250	I	IIIa	100.0%	100.0%	100.0%	100.0%	100.0%	100.0%	100.0%		100.0%	100.0%	100.0%	100.0%	100.0%	100.0%	100.0%	96.8%	96.8%	86.7%	86.7%
NCTC10442	CC8	ST250	I	IIIa	100.0%	100.0%	100.0%	100.0%	100.0%	100.0%	100.0%	100.0%		100.0%	100.0%	100.0%	100.0%	100.0%	100.0%	96.8%	96.8%	86.7%	86.7%
Newman	CC8	ST254	I	IIIa	100.0%	100.0%	100.0%	100.0%	100.0%	100.0%	100.0%	100.0%	100.0%		100.0%	100.0%	100.0%	100.0%	100.0%	96.8%	96.8%	86.7%	86.7%
JCSC6663	CC8	ST254	I	IIIa	100.0%	100.0%	100.0%	100.0%	100.0%	100.0%	100.0%	100.0%	100.0%	100.0%		100.0%	100.0%	100.0%	100.0%	96.8%	96.8%	86.7%	86.7%
JCSC4741	CC8	ST380	I	IIIa	100.0%	100.0%	100.0%	100.0%	100.0%	100.0%	100.0%	100.0%	100.0%	100.0%	100.0%		100.0%	100.0%	100.0%	96.8%	96.8%	86.7%	86.7%
JCSC4746	CC8	ST450	I	IIIa	100.0%	100.0%	100.0%	100.0%	100.0%	100.0%	100.0%	100.0%	100.0%	100.0%	100.0%	100.0%		100.0%	100.0%	96.8%	96.8%	86.7%	86.7%
TSCC26	CC8	ST995	I	IIIa	100.0%	100.0%	100.0%	100.0%	100.0%	100.0%	100.0%	100.0%	100.0%	100.0%	100.0%	100.0%	100.0%		100.0%	96.8%	96.8%	86.7%	86.7%
TSCC12	CC8	ST1252	I	IIIa	100.0%	100.0%	100.0%	100.0%	100.0%	100.0%	100.0%	100.0%	100.0%	100.0%	100.0%	100.0%	100.0%	100.0%		96.8%	96.8%	86.7%	86.7%
M9N	CC88	ST78	III	IIIa	96.3%	96.3%	96.3%	96.3%	96.3%	96.3%	96.3%	96.3%	96.3%	96.3%	96.3%	96.3%	96.3%	96.3%	96.3%		100.0%	86.7%	86.7%
W17	CC88	ST88	III	IIIa	96.3%	96.3%	96.3%	96.3%	96.3%	96.3%	96.3%	96.3%	96.3%	96.3%	96.3%	96.3%	96.3%	96.3%	96.3%	100.0%		86.7%	86.7%
NVAU02070	Minor group	ST1251	II	IIIa	91.1%	91.1%	91.1%	91.1%	91.1%	91.1%	91.1%	91.1%	91.1%	91.1%	91.1%	91.1%	91.1%	91.1%	91.1%	90.1%	90.1%		100.0%
NVAU02080	Minor group	ST1251	II	IIIa	91.1%	91.1%	91.1%	91.1%	91.1%	91.1%	91.1%	91.1%	91.1%	91.1%	91.1%	91.1%	91.1%	91.1%	91.1%	90.1%	90.1%	100.0%	

S2-4. SC type IV

a. D1 region

strain name	CC	ST	agr	SC	93/H44	85/2232	stp28	JCSC6673	TSCC15	AIS2002062	MRSA252	AIS2002054	MR108	85/2082	85/3907	86/961	SH488
93/H44	CC30	ST30	III	IVa		100.0%	100.0%	100.0%	100.0%	100.0%	100.0%	100.0%	100.0%	100.0%	100.0%	100.0%	97.3%
85/2232	CC30	ST30	III	IVa	100.0%		100.0%	100.0%	100.0%	100.0%	100.0%	100.0%	100.0%	100.0%	100.0%	100.0%	97.3%
stp28	CC30	ST30	III	IVa	100.0%	100.0%		100.0%	100.0%	100.0%	100.0%	100.0%	100.0%	100.0%	100.0%	100.0%	97.3%
JCSC6673	CC30	ST30	III	IVa	100.0%	100.0%	100.0%		100.0%	100.0%	100.0%	100.0%	100.0%	100.0%	100.0%	100.0%	97.3%
TSCC15	CC30	ST30	III	IVa	100.0%	100.0%	100.0%	100.0%		100.0%	100.0%	100.0%	100.0%	100.0%	100.0%	100.0%	97.3%
AIS2002062	CC30	ST30	III	IVa	100.0%	100.0%	100.0%	100.0%	100.0%		100.0%	100.0%	100.0%	100.0%	100.0%	100.0%	97.3%
MRSA252	CC30	ST36	III	IVa	100.0%	100.0%	100.0%	100.0%	100.0%	100.0%		100.0%	100.0%	100.0%	100.0%	100.0%	97.3%
AIS2002054	CC30	ST36	III	IVa	100.0%	100.0%	100.0%	100.0%	100.0%	100.0%	100.0%		100.0%	100.0%	100.0%	100.0%	97.3%
MR108	CC30	ST74	III	IVa	100.0%	100.0%	100.0%	100.0%	100.0%	100.0%	100.0%	100.0%		100.0%	100.0%	100.0%	97.3%
85/2082	CC8	ST239	I	IVa	100.0%	100.0%	100.0%	100.0%	100.0%	100.0%	100.0%	100.0%	100.0%		100.0%	100.0%	97.3%
85/3907	CC8	ST239	I	IVa	100.0%	100.0%	100.0%	100.0%	100.0%	100.0%	100.0%	100.0%	100.0%	100.0%		100.0%	97.3%
86/961	CC8	ST239	I	IVa	100.0%	100.0%	100.0%	100.0%	100.0%	100.0%	100.0%	100.0%	100.0%	100.0%	100.0%		97.3%
SH488	CC6	ST6	I	IVb	97.5%	97.5%	97.5%	97.5%	97.5%	97.5%	97.5%	97.5%	97.5%	97.5%	97.5%	97.5%	

b. D2 region

strain name	CC	ST	agr	SC	93/H44	85/2232	stp28	JCSC6673	TSCC15	AIS2002062	MRSA252	AIS2002054	MR108	85/2082	85/3907	86/961	SH488
93/H44	CC30	ST30	III	IVa		99.2%	100.0%	100.0%	100.0%	100.0%	99.2%	99.2%	100.0%	100.0%	100.0%	100.0%	99.2%
85/2232	CC30	ST30	III	IVa	99.7%		99.2%	99.2%	99.2%	99.2%	100.0%	100.0%	99.2%	99.2%	99.2%	99.2%	98.5%
stp28	CC30	ST30	III	IVa	100.0%	99.7%		100.0%	100.0%	100.0%	99.2%	99.2%	100.0%	100.0%	100.0%	100.0%	99.2%
JCSC6673	CC30	ST30	III	IVa	100.0%	99.7%	100.0%		100.0%	100.0%	99.2%	99.2%	100.0%	100.0%	100.0%	100.0%	99.2%
TSCC15	CC30	ST30	III	IVa	100.0%	99.7%	100.0%	100.0%		100.0%	99.2%	99.2%	100.0%	100.0%	100.0%	100.0%	99.2%
AIS2002062	CC30	ST30	III	IVa	100.0%	99.7%	100.0%	100.0%	100.0%		99.2%	99.2%	100.0%	100.0%	100.0%	100.0%	99.2%
MRSA252	CC30	ST36	III	IVa	99.7%	100.0%	99.7%	99.7%	99.7%	99.7%		100.0%	99.2%	99.2%	99.2%	99.2%	98.5%
AIS2002054	CC30	ST36	III	IVa	99.7%	100.0%	99.7%	99.7%	99.7%	99.7%	100.0%		99.2%	99.2%	99.2%	99.2%	98.5%
MR108	CC30	ST74	III	IVa	100.0%	99.7%	100.0%	100.0%	100.0%	100.0%	99.7%	99.7%		100.0%	100.0%	100.0%	99.2%
85/2082	CC8	ST239	I	IVa	100.0%	99.7%	100.0%	100.0%	100.0%	100.0%	99.7%	99.7%	100.0%		100.0%	100.0%	99.2%
85/3907	CC8	ST239	I	IVa	100.0%	99.7%	100.0%	100.0%	100.0%	100.0%	99.7%	99.7%	100.0%	100.0%		100.0%	99.2%
86/961	CC8	ST239	I	IVa	100.0%	99.7%	100.0%	100.0%	100.0%	100.0%	99.7%	99.7%	100.0%	100.0%	100.0%		99.2%
SH488	CC6	ST6	I	IVb	98.7%	98.5%	98.7%	98.7%	98.7%	98.7%	98.5%	98.5%	98.7%	98.7%	98.7%	98.7%	

c. Central region

strain name	CC	ST	agr	SC	93/H44	85/2232	stp28	JCSC6673	TSCC15	AIS2002062	MRSA252	AIS2002054	MR108	85/2082	85/3907	86/961	SH488
93/H44	CC30	ST30	III	IVa		100.0%	100.0%	100.0%	99.5%	100.0%	100.0%	100.0%	100.0%	100.0%	100.0%	100.0%	84.5%
85/2232	CC30	ST30	III	IVa	100.0%		100.0%	100.0%	99.5%	100.0%	100.0%	100.0%	100.0%	100.0%	100.0%	100.0%	84.5%
stp28	CC30	ST30	III	IVa	100.0%	100.0%		100.0%	99.5%	100.0%	100.0%	100.0%	100.0%	100.0%	100.0%	100.0%	84.5%
JCSC6673	CC30	ST30	III	IVa	100.0%	100.0%	100.0%		99.5%	100.0%	100.0%	100.0%	100.0%	100.0%	100.0%	100.0%	84.5%
TSCC15	CC30	ST30	III	IVa	99.8%	99.8%	99.8%	99.8%		99.5%	99.5%	99.5%	99.5%	99.5%	99.5%	99.5%	80.6%
AIS2002062	CC30	ST30	III	IVa	100.0%	100.0%	100.0%	100.0%	99.8%		100.0%	100.0%	100.0%	100.0%	100.0%	100.0%	84.5%
MRSA252	CC30	ST36	III	IVa	100.0%	100.0%	100.0%	100.0%	99.8%	100.0%		100.0%	100.0%	100.0%	100.0%	100.0%	84.5%
AIS2002054	CC30	ST36	III	IVa	100.0%	100.0%	100.0%	100.0%	99.8%	100.0%	100.0%		100.0%	100.0%	100.0%	100.0%	84.5%
MR108	CC30	ST74	III	IVa	100.0%	100.0%	100.0%	100.0%	99.8%	100.0%	100.0%	100.0%		100.0%	100.0%	100.0%	84.5%
85/2082	CC8	ST239	I	IVa	100.0%	100.0%	100.0%	100.0%	99.8%	100.0%	100.0%	100.0%	100.0%		100.0%	100.0%	84.5%
85/3907	CC8	ST239	I	IVa	100.0%	100.0%	100.0%	100.0%	99.8%	100.0%	100.0%	100.0%	100.0%	100.0%		100.0%	84.5%
86/961	CC8	ST239	I	IVa	100.0%	100.0%	100.0%	100.0%	99.8%	100.0%	100.0%	100.0%	100.0%	100.0%	100.0%		84.5%
SH488	CC6	ST6	I	IVb	85.4%	85.4%	85.4%	85.4%	85.4%	85.4%	85.4%	85.4%	85.4%	85.4%	85.4%	85.4%	

S2-5. SC type V

a. D1 region

strain name	CC	ST	agr	SC	No55	Stp-58	Stp-25	C-1C	TSCC17	SH640	01093	AIS2002059
No55	CC51	ST95	IV	Va		100.0%	100.0%	94.4%	94.4%	94.4%	95.1%	95.1%
Stp-58	CC51	ST120	IV	Va	100.0%		100.0%	94.4%	94.4%	94.4%	95.1%	95.1%
Stp-25	CC51	ST121	IV	Va	100.0%	100.0%		94.4%	94.4%	94.4%	95.1%	95.1%
C-1C	CC1	ST188	I	Vb	93.4%	93.4%	93.4%		100.0%	100.0%	99.3%	99.3%
TSCC17	CC1	ST188	I	Vb	93.4%	93.4%	93.4%	100.0%		100.0%	99.3%	99.3%
SH640	CC1	ST188	I	Vb	93.4%	93.4%	93.4%	100.0%	100.0%		99.3%	99.3%
01093	CC8	ST72	I	Vb	93.7%	93.7%	93.7%	99.8%	99.8%	99.8%		100.0%
AIS2002059	CC8	ST72	I	Vb	93.7%	93.7%	93.7%	99.8%	99.8%	99.8%	100.0%	

b. D2 region

strain name	CC	ST	agr	SC	No55	Stp-58	Stp-25	C-1C	TSCC17	SH640	01093	AIS2002059
No55	CC51	ST95	IV	Va		100.0%	100.0%	88.4%	88.4%	88.4%	88.4%	88.4%
Stp-58	CC51	ST120	IV	Va	100.0%		100.0%	88.4%	88.4%	88.4%	88.4%	88.4%
Stp-25	CC51	ST121	IV	Va	100.0%	100.0%		88.4%	88.4%	88.4%	88.4%	88.4%
C-1C	CC1	ST188	I	Vb	91.4%	91.4%	91.4%		100.0%	100.0%	98.5%	98.5%
TSCC17	CC1	ST188	I	Vb	91.4%	91.4%	91.4%	100.0%		100.0%	98.5%	98.5%
SH640	CC1	ST188	I	Vb	91.4%	91.4%	91.4%	100.0%	100.0%		98.5%	98.5%
01093	CC8	ST72	I	Vb	92.5%	92.5%	92.5%	98.5%	98.5%	98.5%		100.0%
AIS2002059	CC8	ST72	I	Vb	92.5%	92.5%	92.5%	98.5%	98.5%	98.5%		

c. Central region

strain name	CC	ST	agr	SC	No55	Stp-58	Stp-25	C-1C	TSCC17	SH640	01093	AIS2002059
No55	CC51	ST95	IV	Va		99.5%	99.5%	75.7%	75.7%	75.7%	75.7%	75.7%
Stp-58	CC51	ST120	IV	Va	99.8%		100.0%	75.7%	75.7%	75.7%	75.7%	75.7%
Stp-25	CC51	ST121	IV	Va	99.8%	100.0%		75.7%	75.7%	75.7%	75.7%	75.7%
C-1C	CC1	ST188	I	Vb	81.9%	81.9%	81.9%		100.0%	100.0%	91.5%	91.5%
TSCC17	CC1	ST188	I	Vb	81.9%	81.9%	81.9%	100.0%		100.0%	91.5%	91.5%
SH640	CC1	ST188	I	Vb	81.9%	81.9%	81.9%	100.0%	100.0%		91.5%	91.5%
01093	CC8	ST72	I	Vb	81.7%	81.6%	81.6%	92.0%	92.0%	92.0%		100.0%
AIS2002059	CC8	ST72	I	Vb	81.7%	81.6%	81.6%	92.0%	92.0%	92.0%	100.0%	

S2-6. SC type VI

a. D1 region

strain name	CC	ST	agr	SC	stp12	IFH556	IFH812	IFH818	RF122	IFH514	IFH568	IFH467	strain M
stp12	Minor group	ST96	III	VIa		99.3%	99.3%	99.3%	99.3%	93.2%	93.2%	93.2%	91.9%
IFH556	CC151	ST705	II	VIb	99.5%		100.0%	100.0%	100.0%	93.9%	93.9%	93.9%	92.6%
IFH812	CC151	ST705	II	VIb	99.5%	100.0%		100.0%	100.0%	93.9%	93.9%	93.9%	92.6%
IFH818	CC151	ST705	II	VIb	99.5%	100.0%	100.0%		100.0%	93.9%	93.9%	93.9%	92.6%
RF122	CC151	ST151	II	VIb	99.5%	100.0%	100.0%	100.0%		93.9%	93.9%	93.9%	92.6%
IFH514	CC97	ST352	I	VIc	97.0%	97.0%	97.0%	97.0%	97.0%		100.0%	100.0%	95.9%
IFH568	CC97	ST352	I	VIc	97.0%	97.0%	97.0%	97.0%	97.0%	100.0%		100.0%	95.9%
IFH467	CC97	ST352	I	VIc	97.0%	97.0%	97.0%	97.0%	97.0%	100.0%	100.0%		95.9%
strain M	Singleton	ST1254	II	VIc	96.6%	96.6%	96.6%	96.6%	96.6%	98.6%	98.6%	98.6%	

b. D2 region

strain name	CC	ST	agr	SC	stp12	IFH556	IFH812	IFH818	RF122	IFH514	IFH568	IFH467	strain M
stp12	Minor group	ST96	III	VIa		94.0%	94.0%	94.0%	94.0%	63.3%	63.3%	63.3%	61.7%
IFH556	CC151	ST705	II	VIb	95.5%		100.0%	100.0%	100.0%	60.2%	60.2%	60.2%	61.7%
IFH812	CC151	ST705	II	VIb	95.5%	100.0%		100.0%	100.0%	60.2%	60.2%	60.2%	61.7%
IFH818	CC151	ST705	II	VIb	95.5%	100.0%	100.0%		100.0%	60.2%	60.2%	60.2%	61.7%
RF122	CC151	ST151	II	VIb	95.5%	100.0%	100.0%	100.0%		60.2%	60.2%	60.2%	61.7%
IFH514	CC97	ST352	I	VIc	69.9%	66.6%	66.6%	66.6%	66.6%		100.0%	100.0%	98.8%
IFH568	CC97	ST352	I	VIc	69.9%	66.6%	66.6%	66.6%	66.6%	100.0%		100.0%	98.8%
IFH467	CC97	ST352	I	VIc	69.9%	66.6%	66.6%	66.6%	66.6%	100.0%	100.0%		98.8%
strain M	Singleton	ST1254	II	VIc	69.0%	68.7%	68.7%	68.7%	68.7%	98.4%	98.4%	98.4%	

c. Central region

strain name	CC	ST	agr	SC	stp12	IFH556	IFH812	IFH818	RF122	IFH514	IFH568	IFH467	strain M
stp12	Minor group	ST96	III	VIa		84.6%	84.6%	84.6%	84.6%	91.5%	91.5%	91.5%	91.6%
IFH556	CC151	ST705	II	VIb	88.8%		100.0%	100.0%	100.0%	86.2%	86.2%	86.2%	92.6%
IFH812	CC151	ST705	II	VIb	88.8%	100.0%		100.0%	100.0%	86.2%	86.2%	86.2%	92.6%
IFH818	CC151	ST705	II	VIb	88.8%	100.0%	100.0%		100.0%	86.2%	86.2%	86.2%	92.6%
RF122	CC151	ST151	II	VIb	88.8%	100.0%	100.0%	100.0%		86.2%	86.2%	86.2%	92.6%
IFH514	CC97	ST352	I	VIc	92.9%	89.9%	89.9%	89.9%	89.9%		100.0%	100.0%	88.4%
IFH568	CC97	ST352	I	VIc	92.9%	89.9%	89.9%	89.9%	89.9%	100.0%		100.0%	88.4%
IFH467	CC97	ST352	I	VIc	92.9%	89.9%	89.9%	89.9%	89.9%	100.0%	100.0%		88.4%
strain M	Singleton	ST1254	II	VIc	92.6%	93.7%	93.7%	93.7%	93.7%	91.6%	91.6%	91.6%	

S2-7. SC type VII

a. D1 region

strain name	CC	ST	agr	SC	MSSA47	MW2	JCSC6076	AIS2002056	91/2619	JCSC4796	TSCC9A	JCSC6665	AIS2002058	JCSC6068	WIS	C-12B	JCSC6908	JCSC6909	AIS2002061	JCSC6664	NVAU02063
MSSA476	CC1	ST1	III	VIIa		100.0%	100.0%	100.0%	100.0%	100.0%	100.0%	98.7%	98.7%	98.7%	98.7%	98.7%	99.3%	99.3%	99.3%	99.3%	98.7%
MW2	CC1	ST1	III	VIIa	100.0%		100.0%	100.0%	100.0%	100.0%	100.0%	98.7%	98.7%	98.7%	98.7%	98.7%	99.3%	99.3%	99.3%	99.3%	98.7%
JCSC6076	CC1	ST1	III	VIIa	100.0%	100.0%		100.0%	100.0%	100.0%	100.0%	98.7%	98.7%	98.7%	98.7%	98.7%	99.3%	99.3%	99.3%	99.3%	98.7%
AIS2002056	CC1	ST1	III	VIIa	100.0%	100.0%	100.0%		100.0%	100.0%	100.0%	98.7%	98.7%	98.7%	98.7%	98.7%	99.3%	99.3%	99.3%	99.3%	98.7%
91/2619	CC1	ST76	III	VIIa	100.0%	100.0%	100.0%	100.0%		100.0%	100.0%	98.7%	98.7%	98.7%	98.7%	98.7%	99.3%	99.3%	99.3%	99.3%	98.7%
JCSC4796	CC1	ST81	III	VIIa	100.0%	100.0%	100.0%	100.0%	100.0%		100.0%	98.7%	98.7%	98.7%	98.7%	98.7%	99.3%	99.3%	99.3%	99.3%	98.7%
TSCC9A	CC1	ST81	III	VIIa	100.0%	100.0%	100.0%	100.0%	100.0%	100.0%		98.7%	98.7%	98.7%	98.7%	98.7%	99.3%	99.3%	99.3%	99.3%	98.7%
JCSC6665	CC45	ST45	I	VIIb	98.9%	98.9%	98.9%	98.9%	98.9%	98.9%	98.9%		100.0%	100.0%	100.0%	100.0%	99.3%	99.3%	99.3%	99.3%	98.7%
AIS2002058	CC45	ST45	I	VIIb	98.9%	98.9%	98.9%	98.9%	98.9%	98.9%	98.9%	100.0%		100.0%	100.0%	100.0%	99.3%	99.3%	99.3%	99.3%	98.7%
JCSC6068	CC45	ST45	I	VIIb	98.9%	98.9%	98.9%	98.9%	98.9%	98.9%	98.9%	100.0%	100.0%		100.0%	100.0%	99.3%	99.3%	99.3%	99.3%	98.7%
WIS	CC45	ST45	I	VIIb	98.9%	98.9%	98.9%	98.9%	98.9%	98.9%	98.9%	100.0%	100.0%	100.0%		100.0%	99.3%	99.3%	99.3%	99.3%	98.7%
C-12B	CC45	ST508	I	VIIb	98.9%	98.9%	98.9%	98.9%	98.9%	98.9%	98.9%	100.0%	100.0%	100.0%	100.0%		99.3%	99.3%	99.3%	99.3%	98.7%
JCSC6908	CC398	ST398	I	VIIb	98.9%	98.9%	98.9%	98.9%	98.9%	98.9%	98.9%	99.6%	99.6%	99.6%	99.6%	99.6%		100.0%	100.0%	100.0%	99.3%
JCSC6909	CC398	ST398	I	VIIb	98.9%	98.9%	98.9%	98.9%	98.9%	98.9%	98.9%	99.6%	99.6%	99.6%	99.6%	99.6%	100.0%		100.0%	100.0%	99.3%
AIS2002061	CC59	ST59	I	VIIc	99.1%	99.1%	99.1%	99.1%	99.1%	99.1%	99.1%	99.3%	99.3%	99.3%	99.3%	99.3%	99.3%	99.3%		100.0%	99.3%
JCSC6664	CC59	ST59	I	VIIc	99.1%	99.1%	99.1%	99.1%	99.1%	99.1%	99.1%	99.3%	99.3%	99.3%	99.3%	99.3%	99.3%	99.3%	100.0%		99.3%
NVAU02063	Singleton	ST1253	I	VIIa/	99.1%	99.1%	99.1%	99.1%	99.1%	99.1%	99.1%	99.3%	99.3%	99.3%	99.3%	99.3%	99.3%	99.3%	99.6%	99.6%	

b. D2 region

strain name	CC	ST	agr	SC	MSSA47	MW2	JCSC6076	AIS2002056	91/2619	JCSC4796	TSCC9A	JCSC6665	AIS2002058	JCSC6068	WIS	C-12B	JCSC6908	JCSC6909	AIS2002061	JCSC6664	NVAU02063
MSSA476	CC1	ST1	III	VIIa		100.0%	100.0%	100.0%	100.0%	100.0%	100.0%	97.7%	97.7%	97.7%	97.7%	97.7%	97.7%	97.7%	96.9%	96.9%	96.1%
MW2	CC1	ST1	III	VIIa	100.0%		100.0%	100.0%	100.0%	100.0%	100.0%	97.7%	97.7%	97.7%	97.7%	97.7%	97.7%	97.7%	96.9%	96.9%	96.1%
JCSC6076	CC1	ST1	III	VIIa	100.0%	100.0%		100.0%	100.0%	100.0%	100.0%	97.7%	97.7%	97.7%	97.7%	97.7%	97.7%	97.7%	96.9%	96.9%	96.1%
AIS2002056	CC1	ST1	III	VIIa	100.0%	100.0%	100.0%		100.0%	100.0%	100.0%	97.7%	97.7%	97.7%	97.7%	97.7%	97.7%	97.7%	96.9%	96.9%	96.1%
91/2619	CC1	ST76	III	VIIa	100.0%	100.0%	100.0%	100.0%		100.0%	100.0%	97.7%	97.7%	97.7%	97.7%	97.7%	97.7%	97.7%	96.9%	96.9%	96.1%
JCSC4796	CC1	ST81	III	VIIa	100.0%	100.0%	100.0%	100.0%	100.0%		100.0%	97.7%	97.7%	97.7%	97.7%	97.7%	97.7%	97.7%	96.9%	96.9%	96.1%
TSCC9A	CC1	ST81	III	VIIa	100.0%	100.0%	100.0%	100.0%	100.0%	100.0%		97.7%	97.7%	97.7%	97.7%	97.7%	97.7%	97.7%	96.9%	96.9%	96.1%
JCSC6665	CC45	ST45	I	VIIb	96.7%	96.7%	96.7%	96.7%	96.7%	96.7%	96.7%		100.0%	100.0%	100.0%	100.0%	100.0%	100.0%	99.2	99.2%	98.4%
AIS2002058	CC45	ST45	I	VIIb	96.7%	96.7%	96.7%	96.7%	96.7%	96.7%	96.7%	100.0%		100.0%	100.0%	100.0%	100.0%	100.0%	99.2	99.2%	98.4%
JCSC6068	CC45	ST45	I	VIIb	96.7%	96.7%	96.7%	96.7%	96.7%	96.7%	96.7%	100.0%	100.0%		100.0%	100.0%	100.0%	100.0%	99.2	99.2%	98.4%
WIS	CC45	ST45	I	VIIb	96.7%	96.7%	96.7%	96.7%	96.7%	96.7%	96.7%	100.0%	100.0%	100.0%		100.0%	100.0%	100.0%	99.2	99.2%	98.4%
C-12B	CC45	ST508	I	VIIb	96.7%	96.7%	96.7%	96.7%	96.7%	96.7%	96.7%	100.0%	100.0%	100.0%	100.0%		100.0%	100.0%	99.2	99.2%	98.4%
JCSC6908	CC398	ST398	I	VIIb	96.9%	96.9%	96.9%	96.9%	96.9%	96.9%	96.9%	99.7%	99.7%	99.7%	99.7%	99.7%		100.0%	99.2	99.2%	98.4%
JCSC6909	CC398	ST398	I	VIIb	96.9%	96.9%	96.9%	96.9%	96.9%	96.9%	96.9%	99.7%	99.7%	99.7%	99.7%	99.7%	100.0%		99.2	99.2%	98.4%
AIS2002061	CC59	ST59	I	VIIc	97.7%	97.7%	97.7%	97.7%	97.7%	97.7%	97.7%	98.7%	98.7%	98.7%	98.7%	98.7%	99.0%	99.0%		100.0%	99.2%
JCSC6664	CC59	ST59	I	VIIc	97.7%	97.7%	97.7%	97.7%	97.7%	97.7%	97.7%	98.7%	98.7%	98.7%	98.7%	98.7%	99.0%	99.0%	100.0%		99.2%
NVAU02063	Singleton	ST1253	I	VIIa/	97.4%	97.4%	97.4%	97.4%	97.4%	97.4%	97.4%	98.5%	98.5%	98.5%	98.5%	98.5%	98.7%	98.7%	99.7%	99.7%	

c. Central region

strain name	CC	ST	agr	SC	MSSA47	MW2	JCSC6076	AIS2002056	91/2619	JCSC4796	TSCC9A	JCSC6665	AIS2002058	JCSC6068	WIS	C-12B	JCSC6908	JCSC6909	AIS2002061	JCSC6664	NVAU02063
MSSA476	CC1	ST1	III	VIIa		100.0%	100.0%	100.0%	100.0%	100.0%	100.0%	78.4%	78.4%	78.4%	78.4%	78.4%	78.4%	78.4%	83.5%	83.5%	89.9%
MW2	CC1	ST1	III	VIIa	100.0%		100.0%	100.0%	100.0%	100.0%	100.0%	78.4%	78.4%	78.4%	78.4%	78.4%	78.4%	78.4%	83.5%	83.5%	89.9%
JCSC6076	CC1	ST1	III	VIIa	100.0%	100.0%		100.0%	100.0%	100.0%	100.0%	78.4%	78.4%	78.4%	78.4%	78.4%	78.4%	78.4%	83.5%	83.5%	89.9%
AIS2002056	CC1	ST1	III	VIIa	100.0%	100.0%	100.0%		100.0%	100.0%	100.0%	78.4%	78.4%	78.4%	78.4%	78.4%	78.4%	78.4%	83.5%	83.5%	89.9%
91/2619	CC1	ST76	III	VIIa	100.0%	100.0%	100.0%	100.0%		100.0%	100.0%	78.4%	78.4%	78.4%	78.4%	78.4%	78.4%	78.4%	83.5%	83.5%	89.9%
JCSC4796	CC1	ST81	III	VIIa	100.0%	100.0%	100.0%	100.0%	100.0%		100.0%	78.4%	78.4%	78.4%	78.4%	78.4%	78.4%	78.4%	83.5%	83.5%	89.9%
TSCC9A	CC1	ST81	III	VIIa	100.0%	100.0%	100.0%	100.0%	100.0%	100.0%		78.4%	78.4%	78.4%	78.4%	78.4%	78.4%	78.4%	83.5%	83.5%	89.9%
JCSC6665	CC45	ST45	I	VIIb	82.9%	82.9%	82.9%	82.9%	82.9%	82.9%	82.9%		100.0%	100.0%	100.0%	100.0%	100.0%	100.0%	88.9%	88.9%	76.4%
AIS2002058	CC45	ST45	I	VIIb	82.9%	82.9%	82.9%	82.9%	82.9%	82.9%	82.9%	100.0%		100.0%	100.0%	100.0%	100.0%	100.0%	88.9%	88.9%	76.4%
JCSC6068	CC45	ST45	I	VIIb	82.9%	82.9%	82.9%	82.9%	82.9%	82.9%	82.9%	100.0%	100.0%		100.0%	100.0%	100.0%	100.0%	88.9%	88.9%	76.4%
WIS	CC45	ST45	I	VIIb	82.9%	82.9%	82.9%	82.9%	82.9%	82.9%	82.9%	100.0%	100.0%	100.0%		100.0%	100.0%	100.0%	88.9%	88.9%	76.4%
C-12B	CC45	ST508	I	VIIb	82.9%	82.9%	82.9%	82.9%	82.9%	82.9%	82.9%	100.0%	100.0%	100.0%	100.0%		100.0%	100.0%	88.9%	88.9%	76.4%
JCSC6908	CC398	ST398	I	VIIb	83.4%	83.4%	83.4%	83.4%	83.4%	83.4%	83.4%	99.5%	99.5%	99.5%	99.5%	99.5%		100.0%	88.9%	88.9%	76.4%
JCSC6909	CC398	ST398	I	VIIb	83.4%	83.4%	83.4%	83.4%	83.4%	83.4%	83.4%	99.5%	99.5%	99.5%	99.5%	99.5%	100.0%		88.9%	88.9%	76.4%
AIS2002061	CC59	ST59	I	VIIc	88.8%	88.8%	88.8%	88.8%	88.8%	88.8%	88.8%	89.4%	89.4%	89.4%	89.4%	89.4%	89.6%	89.6%		100.0%	85.6%
JCSC6664	CC59	ST59	I	VIIc	88.8%	88.8%	88.8%	88.8%	88.8%	88.8%	88.8%	89.4%	89.4%	89.4%	89.4%	89.4%	89.6%	89.6%	100.0%		85.6%
NVAU02063	Singleton	ST1253	I	VIIa/	92.4%	92.4%	92.4%	92.4%	92.4%	92.4%	92.4%	82.2%	82.2%	82.2%	82.2%	82.2%	82.2%	82.2%	91.3%	91.3%	

S2-8. SC type VIII

a. D1 region

strain name	CC	ST	agr	SC	Ku	NVAU02071	NVAU02081
Ku	Minor group	ST10	NT	VIIIa		92.6%	92.6%
NVAU02071	CC20	ST20	I	VIIIb	94.9%		100.0%
NVAU02081	CC20	ST20	I	VIIIb	94.9%	100.0%	

b. D2 region

strain name	CC	ST	agr	SC	Ku	NVAU02071	NVAU02081
Ku	Minor group	ST10	NT	VIIIa		93.8%	93.8%
NVAU02071	CC20	ST20	I	VIIIb	93.6%		93.8%
NVAU02081	CC20	ST20	I	VIIIb	93.6%	100.0%	

c. Central region

strain name	CC	ST	agr	SC	Ku	NVAU02071	NVAU02081
Ku	Minor group	ST10	NT	VIIIa		70.5%	70.5%
NVAU02071	CC20	ST20	I	VIIIb	77.3%		100.0%
NVAU02081	CC20	ST20	I	VIIIb	77.3%	100.0%	

S2-9. SC type X

a. D1 region

strain name	CC	ST	agr	SC	19	JCSC6074	JCSC6666
19	CC15	ST15	II	Xa		97.3%	97.3%
JCSC6074	Minor group	ST140	IV	Xb	97.8%		100.0%
JCSC6666	Minor group	ST140	IV	Xb	97.8%	100.0%	

b. D2 region

strain name	CC	ST	agr	SC	19	JCSC6074	JCSC6666
19	CC15	ST15	II	Xa		93.8%	93.8%
JCSC6074	Minor group	ST140	IV	Xb	96.4%		100.0%
JCSC6666	Minor group	ST140	IV	Xb	96.4%	100.0%	

c. Central region

strain name	CC	ST	agr	SC	19	JCSC6074	JCSC6666
19	CC15	ST15	II	Xa		78.9%	78.9%
JCSC6074	Minor group	ST140	IV	Xb	86.3%		100.0%
JCSC6666	Minor group	ST140	IV	Xb	86.3%	100.0%	

S2-10. SC type XI

a. D1 region

strain name	CC	ST	agr	SC	JCSC607	JCSC6667	JCSC6671	JCSC6674	JCSC6906	JCSC6907	JCSC6669
JCSC6075	CC22	ST22	I	XIa		100.0%	100.0%	100.0%	100.0%	100.0%	95.9%
JCSC6667	CC22	ST22	I	XIa	100.0%		100.0%	100.0%	100.0%	100.0%	95.9%
JCSC6671	CC22	ST22	I	XIa	100.0%	100.0%		100.0%	100.0%	100.0%	95.9%
JCSC6674	CC22	ST22	I	XIa	100.0%	100.0%	100.0%		100.0%	100.0%	95.9%
JCSC6906	CC22	ST22	I	XIa	100.0%	100.0%	100.0%	100.0%		100.0%	95.9%
JCSC6907	CC22	ST22	I	XIa	100.0%	100.0%	100.0%	100.0%	100.0%		95.9%
JCSC6669	CC182	ST182	I	XIb	93.8%	93.8%	93.8%	93.8%	93.8%	93.8%	

b. D2 region

strain name	CC	ST	agr	SC	JCSC607	JCSC6667	JCSC6671	JCSC6674	JCSC6906	JCSC6907	JCSC6669
JCSC6075	CC22	ST22	I	XIa		100.0%	100.0%	100.0%	100.0%	100.0%	91.7%
JCSC6667	CC22	ST22	I	XIa	100.0%		100.0%	100.0%	100.0%	100.0%	91.7%
JCSC6671	CC22	ST22	I	XIa	100.0%	100.0%		100.0%	100.0%	100.0%	91.7%
JCSC6674	CC22	ST22	I	XIa	100.0%	100.0%	100.0%		100.0%	100.0%	91.7%
JCSC6906	CC22	ST22	I	XIa	100.0%	100.0%	100.0%	100.0%		100.0%	91.7%
JCSC6907	CC22	ST22	I	XIa	100.0%	100.0%	100.0%	100.0%	100.0%		91.7%
JCSC6669	CC182	ST182	I	XIb	92.0%	92.0%	92.0%	92.0%	92.0%	92.0%	

c. Central region

strain name	CC	ST	agr	SC	JCSC607	JCSC6667	JCSC6671	JCSC6674	JCSC6906	JCSC6907	JCSC6669
JCSC6075	CC22	ST22	I	XIa		100.0%	100.0%	100.0%	100.0%	100.0%	78.6%
JCSC6667	CC22	ST22	I	XIa	100.0%		100.0%	100.0%	100.0%	100.0%	78.6%
JCSC6671	CC22	ST22	I	XIa	100.0%	100.0%		100.0%	100.0%	100.0%	78.6%
JCSC6674	CC22	ST22	I	XIa	100.0%	100.0%	100.0%		100.0%	100.0%	78.6%
JCSC6906	CC22	ST22	I	XIa	100.0%	100.0%	100.0%	100.0%		100.0%	78.6%
JCSC6907	CC22	ST22	I	XIa	100.0%	100.0%	100.0%	100.0%	100.0%		78.6%
JCSC6669	CC182	ST182	I	XIb	81.1%	81.1%	81.1%	81.1%	81.1%	81.1%	
